# A simulation approach for collaborative humanitarian aid distribution management: the case of Bogotá city

**DOI:** 10.1016/j.heliyon.2022.e11465

**Published:** 2022-11-15

**Authors:** Diana C. Guzmán-Cortés, Leonardo Gonzaléz-Rodríguez, Carlos Franco, William J. Guerrero

**Affiliations:** a Research Group in Logistics Systems. Faculty of Engineering, Universidad de La Sabana Chía, Colombia; b Faculty of Engineering, Universidad El Bosque Bogotá, Colombia; c Universidad Del Rosario, School of Management and Business, Bogotá, Colombia; d Universidad Del Magdalena, Santa Marta, Colombia

**Keywords:** Humanitarian supply chain, Collaboration, System dynamics, Disaster management, Collaborative planning

## Abstract

Collaboration mechanisms in humanitarian supply chains increase efficiency levels in post-disaster relief operations. The scientific literature on the topic is mostly based on qualitative approaches to identify the barriers and benefits of collaborative relationships between stakeholders. Thus, this paper proposes the use of system dynamics simulation to evaluate the impact on the average response time of aid delivery resulting from implementing different collaborative strategies. In this sense, collaborative strategies are designed for humanitarian operations in the immediate response phase considering elements such as information exchange, shared resources, and infrastructure. To design and assess the impact of these collaborative strategies in a disaster scenario, we analyze the case of the city of Bogotá-Colombia and the surrounding cities, we have used public information for building and tuning the parameters of the simulation model. The simulation model results allow us to identify the main factors that reduce the average response times to serve the affected population. It can be concluded that the strategy which includes sharing resources and infrastructure, exchanging information, and the creation of joint knowledge has a greater improvement in response times becoming the best policy for disaster management given the results obtained in a collaborative planning environment.

## 1 Introduction

According to the annual report of the Centre for Research On the Epidemiology of disasters (2022) in 2021, there were 432 natural disasters, a number over the average of the last ten years (357 events in 2001–2020 annual average). Also, the report shows that 10,492 people died from these events, the affected people were calculated at 101.8 million and the economic loss was estimated at US$ 252 billion. Therefore, humanitarian organizations have important pressures to increase the cost-efficiency of their operations achieving their goals under a limited budget. According to Tatham and Kovács (2010) it can be concluded that logistics are the greater expenses in humanitarian operations which can be up to 80% of the total costs. Then, this research is dedicated to analyzing collaboration mechanisms in a humanitarian supply chain that optimize the efficiency of the operations.

According to (Pearce (2000)), the definition of a disaster is related to a non-routine event that overwhelms the capacity response and threatens the stability of the affected region in different dimensions such as social, ecological, economic, and political. Therefore, a disaster cannot be faced only with local resources, differently from an emergency that can be managed with local resources (Drabek, 1996). Another definition of disaster is given by Van Wassenhove, (2006) for whom a disaster can be defined as a disorder that affects the goals of a physical system. Then, a natural event can be categorized as a natural disaster when it occurs in populated places, there is infrastructure damage, economic loss, and people without access to satisfy their basic needs in a state of suffering (da Costa et al., 2012). Further, a catastrophe is defined as a disaster that exceeds the ability of state agencies, local agencies and volunteers to provide the assistance such as food, shelter and medical care to the victims within the first 12–24 h after the event (U.S Government Accountability Office, 2006).

Humanitarian logistics is defined as the processes and systems involved in the mobilization of people, resources, skills and knowledge to help vulnerable people affected by disasters. The aim of humanitarian operations is to serve population affected by disasters with the right aid on the right time, in the right place and in the right amount (Balcik et al., 2016).

Given the increasing number of natural disasters, the humanitarian logistics or humanitarian operations have received special interest from researchers and practitioners for developing appropriate tools for planning and controlling operations. This interest is supported by the idea that these disasters affect thousands of lives, generates millions of people affected and the damages could be reduced if improvements were implemented into the logistics operations of the humanitarian systems (da Costa et al., 2012).

In humanitarian logistics process the complexity is influenced by the interaction of different stakeholders (NGOs, governments, military forces, among others) (Heaslip et al., 2012). The Pan American Health Organization (PAHO) identified as actors of the humanitarian logistics system the local governments, international governments and different types of agencies. Oloruntoba and Gray (2006) identify governments as donors and Holguín-Veras et al. (2012) propose to integrate aid efforts with local social networks called Collaborative Aid Networks (CAN).

This large number of actors that come together to provide assistance to the affected population can generate collaboration and coordination problems in the humanitarian supply chain. The individual objectives of the different organizations involved in the humanitarian operations not always lead to integrated and coordinated efforts, and therefore, humanitarian action could be invalidated (Balcik et al., 2010a; Carroll and Neu, 2009; Chandes and Paché, 2009). Therefore, one aspect to achieve success in an humanitarian operations is related to the collaboration and coordination (Chandes and Paché, 2009; Dorasamy et al., 2013). Without proper coordination and collaboration mechanisms, the actors could compete for the resources, and as a consequence, response times can increase. Therefore, the well-being of victims and affected population is decreased. According to Váncza et al. (2011), it is possible to generate collaboration if there is exchange of information between actors involved.

If we analyze the collaboration in supply chains, it is related to plan and execute operations when two of more different organizations are involved (Simatupang and Sridharan, 2002). When companies collaborate they can share risks and get complementary resources (Park et al., 2004), reduce the costs, improve productivity (Kalwani and Narayandas, 1995), and improve benefits and competitive advantages over time (Mentzer et al., 2000). While recent literature on collaborative humanitarian supply chain operations is vast and real cases of collaboration are documented in the literature, for example the case of UPS and UNHCR in operations in South Sudan (Bealt et al., 2016), most of the research is based on qualitative approaches and the assessment on barriers and benefits are mostly based on interviews and surveys to make perception analysis of stakeholders (Polater, 2020). Thus, there is still little evidence on the magnitude of the benefits of collaboration, an assessment of outcomes, a quantitative comparison between collaboration strategies, and studies to understand the best collaboration strategy to minimize response times and operational costs in disaster relief. This article aims to fill this gap.

Also, it is important to take into account that logistics in humanitarian operations is characterized by high levels of uncertainty, risk, and emergency. This is a significant difference with the traditional commercial logistics setting (Gatignon et al., 2010). Hence, significant research of designing agile humanitarian operations and its supply chain given its particular features is required (Kovács and Spens, 2011). In this way, some works have been developed in the humanitarian context applying different disciplines that contribute to improve the logistics operation. For example, there are similarities between humanitarian and military operations such as the diversity of participants, funding and financial structure, high level of uncertainty, limited resources, politicized environments and instability in the network (Balcik et al., 2010). In this sense, concepts from the focused logistics theory can be incorporated in the design process of collaborative logistics strategies in humanitarian operations. This theory highlights the importance of coordination and collaboration between different echelons as a crucial aspect in humanitarian operations. The key concepts to consider are: interoperability, that is the property that allows all actors to know the limitations and capacities of each other; inter-institutional operations that are performed no matter the cultural differences or unaligned priorities; and finally, command and control structures that include planning, coordination, management and control of forces and operations (Defense, 2000).

To understand these complexities, Guzmán-Cortés et al. (2019) provide adaptions of project management modelling using system dynamics (SD). They propose a tool to improve the planning process of humanitarian operations, considering quantitative and qualitative variables, associated to the concept of collaboration to improve the humanitarian response.

In fact, given the complexity of humanitarian systems, the use of SD simulation models is appropriate since it is intended to model the complexity of the subsystems, the structures that compose the entire system, the feedback processes, the unpredictability of the requirements for humanitarian aids, and the structural delays. Thus, SD models simulate and evaluate policies in the design of strategies and decision-making processes. Besiou et al. (2014) have assessed the suitability of the SD methodology to assist decision-making in the vehicle supply chains in support of humanitarian field operations. Nevertheless, despite the potential of the modelling approach, according to Altay and Green (2006) only 1.8% of 109 articles reviewed used this tool and according to Galindo and Batta (2013) only 1.3% of 155 articles reviewed.

Therefore, the contributions of this paper are threefold: first, to propose collaborations strategies of aid delivery. Second, to assess the impact on average response times, using system dynamics modelling approach. Third, we present the case of the aid management system in Bogotá, Colombia, and the metropolitan region to test our model. Thus, our approach allows to compare collaborative logistics strategies in terms of the average response time for delivering kits during the immediate response phase after a disaster. It means, from the moment the disaster occurs until the first week after. The base simulation model is built with the idea that all actors involved share infrastructure, but no information is shared. Then, different policies are tested to analyze the average response times of the operations, considering information exchange without shared infrastructure, shared infrastructure and information exchange about the delivered aids, shared infrastructure, and information exchange about the required aids.

This paper is organized as follows: In section 2, a literature review about humanitarian logistics focused on collaborative strategies is presented followed by the methodology developed for this study in Section 3. In Section 4 the results and analysis are presented and finally in Section 5 the conclusions are presented.

## 2 Literature review

This section analyzes literature related to aid distribution in humanitarian operations and planning methodologies in two subsections: section 2.1 reviews the elements of collaboration, coordination, and cooperation considered in previous studies, and section 2.2 reviews the use of System Dynamics (SD) in the humanitarian relief context. In the first subsection, the keywords used were (humanitarian logistics or humanitarian supply chain) and (coordination or collaboration or coordination) with the aim to analyze how these elements has been addressed in the literature. The second part was dedicated to the use of system dynamics into humanitarian contexts; therefore, the keywords used were (humanitarian logistics or humanitarian supply chain) and (system dynamics) with the objective of finding how the system dynamics methodology is applied in the humanitarian context and what type of models has been built. Thus, the objective of this is to present the related studies to identify the differences and contributions of our research.

### 2.1 Coordination, cooperation, and collaboration in aid distribution for humanitarian logistics

Previous reviews analyze the humanitarian operations challenges. For example, Behl and Dutta (2019) present a literature review of articles published between 2011-2017 with 892 articles were 78 are dedicated to optimization, agility, coordination and/or collaboration. One of the main findings of this review is the fact that humanitarian supply chain/humanitarian logistics is an emerging field with a diversity of techniques and research areas. Another review is developed by (Banomyong et al., 2019) who review articles related to mitigation, preparedness, response and recovery phases published between 2005 and 2016. The methodology to identify the articles is based on citation networks. They conclude that most articles are focused on mitigation and preparation. In this sense, our proposal is focused on the immediate response phase.

Oftentimes the terms collaboration, cooperation, and coordination are considered as synonyms. The term collaboration in supply chain management is related to the fact of planning and executing synchronized operations between two or more organizations (Simatupang and Sridharan, 2002). Therefore, collaboration in humanitarian logistics can be defined as the joint work between suppliers, donors, ONGs, military forces, governments, UN, among others, to improve the response times and increasing the welfare of the affected population (Ertem et al., 2010).

Tatham and Kovács (2010) present a theoretical framework related to coordination in humanitarian supply chains. They study the importance of information sharing and coordination and its impact of swift trust. For this purpose, the authors develop a survey for disaster response organizations in India analyzing 187 papers to conclude how the trust in sharing information and cooperation between actors of the chain can lead decision makers to design humanitarian operations.

An experiential learning approach focused on collaboration in humanitarian supply chains is proposed by (Wagner and Thakur-Weigold, 2018). In this study, authors analyze the challenges of collaboration with international agencies and the directly and indirectly associated supply chains. The conclusion of the study is that the knowledge of commercial logistics and supply chain should be applied to humanitarian logistics given the benefits that can be achieved in terms of improving the efficiency, effectiveness, and waste reduction.

A study that analyzes the coordination in humanitarian contexts applied to evacuation systems is presented by (Sopha et al., 2019). The authors develop an agent-based simulation model including the human behavior as a key element for the evacuation process integrating coordination elements and its relationship with the total unmet demand. The conclusions of the experimentation support the idea of including the collaboration and information sharing components as factors of analysis given that their results show the importance of these elements for satisfying the requirements.

Velasquez et al. (2019) develop a mathematical optimization model considering the cooperation between different agencies and the prepositioning of relief items. In this sense, a central agency or government can share resources with other agencies to improve the efficiency of the aid distribution. The uncertainty in demand and link disruptions are considered to minimize the total demand-weighted distance from distribution centers to dispensing locations. Finally, authors propose a robust optimization model and a heuristic to analyze humanitarian operations.

Similarly, Coskun, Elmaghraby, Karaman and Salman (2019) studied the benefits of cooperation between humanitarian agencies focused on stocking decisions. The proposed approach is based on the incorporation of disasters risk into the Newsvendor problem of two different humanitarian agencies. This allows to optimize the stock levels in case of the occurrence of a major disaster. Their proposal is based on the numerical solution of the Nash equilibrium based on real case disasters.

A post-disaster study is presented by (Maharjan and Hanaoka, 2019) with a mathematical model for locating hubs for disaster relief operations. The cooperation and coordination aspects are analyzed in the use of regional logistics hubs. Further, they propose fuzzy weights to include the uncertainty and ambiguity associated with the disaster response phase. A comparative study is performed to evaluate the proposed method with a real case of disaster that considers multiple actors in the decision-making process.

A cooperation and competition model between NGO’s in humanitarian supply chains with government intervention is studied by (Fathalikhani et al., 2020). They optimize the governmental intervention in the relief operation and study how the coopetition and competition of different NGO’s affect the governmental policies. Two different criteria were proposed to test the results: maximization of social welfare and minimization of budget consumption. They show the impact on the performance of the humanitarian operation of these criteria. Four different mathematical models are implemented to identify the interaction between the NGO’s and its impact on the performance metrics concluding that more success of the governmental policies requires more cooperation of the NGO’s and there is an increase in welfare when the coopetition of NGO’s is in place. These results are directly associated to our study because we are measuring the impact of cooperation between different actors in the welfare of population using simulation techniques.

Florian et al. (2021) propose a framework for public-private emergency collaboration based on concepts of logistics and game theory. They present the role of public and private actors in the emergency management characterizing the different types of actors in humanitarian aid, then they introduce the logistics challenges including an analysis of collaboration, interaction, capabilities, strategy, and motivation between the different actors. Finally, from the perspective of game theory, propose a model that gives insights into the motivation and incentives of the partnership as part of the collaborative humanitarian process. In the same sense, Carland et al. (2018) develop an study to identify the agents’ preferences in the humanitarian supply chains. The proposal is based on a multi-attribute value analysis to identify these preferences and to generate value in the outcomes of the humanitarian operations. This proposal is evaluated in a real case in Uganda.

There are also studies related to risk management focused on the technological side or the community perspective. For example Iqbal et al. (2021) emphasize the lack of collaboration between different disciplines for disaster management where authors proposed a framework to support the review process of technology-based contributions in disasters. On the other hand, Nugraheni et al. (2022) developed a structural equation model to evaluate and propose policies programs for flood mitigation. In a similar sense, Ejemeyovwi et al. (2022) developed an analysis through the use of public documents of Nigeria, evaluating the practices and policies of disaster risk management.

Thus, this analysis of the literature shows a gap regarding how to formally understand the impact of the collaboration strategies between several actors in the disaster relief operations using mathematical modelling, as highlighted by (Fosso Wamba, 2020). Our article contributes to fill this gap. We have proposed a methodology to create and evaluate collaborative strategies. The definition of the strategies is made by including characteristics of collaboration-cooperation found in the literature that led us to identify different related factors. With this information, we analyze different types of strategies, not only one as it is common on different studies. Moreover, we have proposed the use of a simulation model based on system dynamics combined with project management techniques for modeling the system. Finally, most of the papers are focused on the preparation phase (Banomyong et al., 2019) meanwhile our approach is focused on the immediate response phase to evaluate the response times to attend the affected population.

Further, little documented knowledge is found on the Latin American context of disaster relief according to Nagendra et al. (2020). Then, our work also fills this gap since we are focused on the analysis of the Colombian context.

### 2.2 System dynamics in humanitarian relief context

According to Altay and Green (2006) in their literature review about OR/MS research in disaster operations management, 1.8% of a total of 109 papers use SD as a modeling technique. This review was updated by (Galindo and Batta, 2013) supporting the previous statement, they found that 1.3% of 155 reviewed papers used this modeling technique.

Meanwhile, Besiou, Stapleton and Van Wassenhove (2011) identify the suitability of SD methodology as a tool for supporting decision makers to understand the impact of their decisions on humanitarian operations performance considering the multiplicity of stakeholders, uncertainty, and complexity. Besiou and Van Wassenhove (2015) recognize SD as a necessary method to understand the behavior of the whole system and achieve a holistic view (Besiou and Van Wassenhove, 2020). Thus, SD allows getting insights into the drivers of the humanitarian operations.

In the same way, Behl and Dutta (2019) highlight SD modeling as a prominent research area in the humanitarian supply chain, because it involves a cause and effect analysis, allowing to design policies and strategies. The authors propose this technique given the complexity of the disaster operations and the uncertainty associated with the demand, infrastructure, and accessibility issues in the humanitarian contexts.

In addition, Mishra et al. (2019) present a literature review focused on disaster management simulation modeling. The authors identify that 42 of 100 selected papers use SD as a modeling method. 21 of them are focused on disaster risk identification and assessment. 15 papers deal with disaster prevention and recovery schemes. A contribution of our study is to propose system dynamics as a modeling technique for logistics operations in the immediate response phase, specifically for the aid delivery operation, as opposed to previous papers dealing with mitigation, preparedness, or recovery phases.

Aid delivery operations are particularly interesting since they have high complexity, a multiplicity of actors and interactions, the operations present uncertainty, delays, and feedback loops. Thus, we decide to use system dynamics as the appropriate modeling tool for the purpose of this study since it allows us to model the system, understanding the behavior, and evaluating the performance of different proposed collaborative strategies.

This idea is supported by (Besiou and Van Wassenhove, 2021), who argue that system dynamics tools allow us to represent and understand the complexity of the humanitarian supply chain. Nevertheless, the full potential of SD in humanitarian logistics is not yet attained (Besiou and Van Wassenhove, 2021). analyze the SD publications in a specialized journal in humanitarian logistics, identifying that most of the publications present a conceptual model and causal loop diagram, and only three works present a simulation model (Besiou et al., 2011) (Diedrichs et al., 2016) (Allahi et al., 2021). In our case, we analyze the relationship between the relevant variables through a causal loop diagram and we build a simulation model to evaluate the impact of different collaborative strategies in the response time.

Some previous works using system dynamics in a disaster relief context are presented. First, Besiou et al. (2014) evaluate centralization, decentralization, or hybridization policies for vehicle supply chains that support humanitarian operations (Pedraza Martinez et al., 2011). studied the vehicle procurement as a tactical decision with a middle-term replacement policy. The authors evaluate and compare the results between the model and the actual policies. Peng et al. (2014) propose a systems dynamic model for post-seismic inventory and logistics planning considering dynamic road capacity, dynamic transport delay, and dynamic information delay. The authors conclude that information sharing is a key element to improve the logistics operations specifically when it is shared between actors in the affected area and the central decision-makers. In our case, we include the information sharing in three proposed strategies for collaborative planning. This key factor affects the humanitarian aid replenishment decisions made by each stakeholder and the unsatisfied demand. Thus, we model a reverse information flow to stop the procurement process, and the planning process to estimate the amount of required aids in a mathematical model to make decisions collaboratively.

Diedrichs et al. (2016) present a system dynamic model to study the role of information sharing in the transportation of commodities from suppliers to disaster points in a network of limited capacity, considering the number of deaths and the monetary cost as indicators of performance. The authors conclude that information sharing implies a cost and there are barriers such as political interests, donor requirements, distant geographical origin, styles of management, and administrative structures. Also, they highlight that Information sharing could involve coordination, cooperation, and collaboration if it is more than the transmission of data and the decision-making is done with the interactions of stakeholders.

Powell et al. (2016) use qualitative system dynamics focused on risk identification and assessment for disaster preparedness, through the construction of causal loops guided by a focus group of experts. They propose this methodology for improving risk understanding by system knowledge and develop it in a case of a flood threat for an electricity station. Also (da Silva et al., 2020), are focused on risk mitigation to identify the main factors that affect the effectiveness of early warning systems through a qualitative system analysis with causal loops from the knowledge of experts. In our study, we also present causal loops to understand the interactions between the main variables in the aid delivery system and levels of collaboration.

Sutley and Hamideh (2018) present a system dynamic model for the post-disaster long-term housing recovery process. The model integrates multi-disciplinary areas such as structural engineering, urban planning, sociology among others with the aim of evaluating the policies that improve the recovery process and reduce inequalities. In contrast with this work, we are focused on short term decisions about logistics operations which involve local and global procurement activities, inventory management and distribution.

An additional study in housing recovery is presented by (Diaz et al., 2020). The authors propose a system dynamic model for the housing recovery problem in a hypothetical scenario of a hurricane, considering short-term repair, long-term reconstruction, and quick fixes. Similar to our study, the model considers material flow and labor flow as key resources. In our case we consider two specific supply chains for each independent flow, and we also include a labor productivity factor considering the number of kits that each staff member can assemble and deliver.

We conclude from this review that, to the best of our knowledge, the reviewed studies do not include key elements of collaborative logistics. Some of them include information-sharing just as an information flow. Therefore, in our study, we assess the influence of different collaborative logistics strategies with the aim to estimate the impact on the average response time. In our work we analyze the relationship between variables using a causal loop diagram and we build a simulation model.

Finally, the research question addressed in this work is how collaborative strategies can affect the average response time in the immediate response phase given the context of a sudden-onset natural disaster. Further, the strategies include relevant elements such as the number of actors involved, sharing of information, resources, and infrastructure between stakeholders.

## 3 Methodology

The methodology used in this research is composed by two phases. The first phase is dedicated to the design of collaborative strategies in the disaster relief operations. In this case, we are based on previous works in the literature that consider collaborative strategies in humanitarian operations (Simatupang and Sridharan, 2002) with the aim of providing an adequate response to the affected population in the right place, at the right time and in the amount required for managing disasters through the integration and exchange of resources and information (Richey et al., 2012).

**Figure 1:**
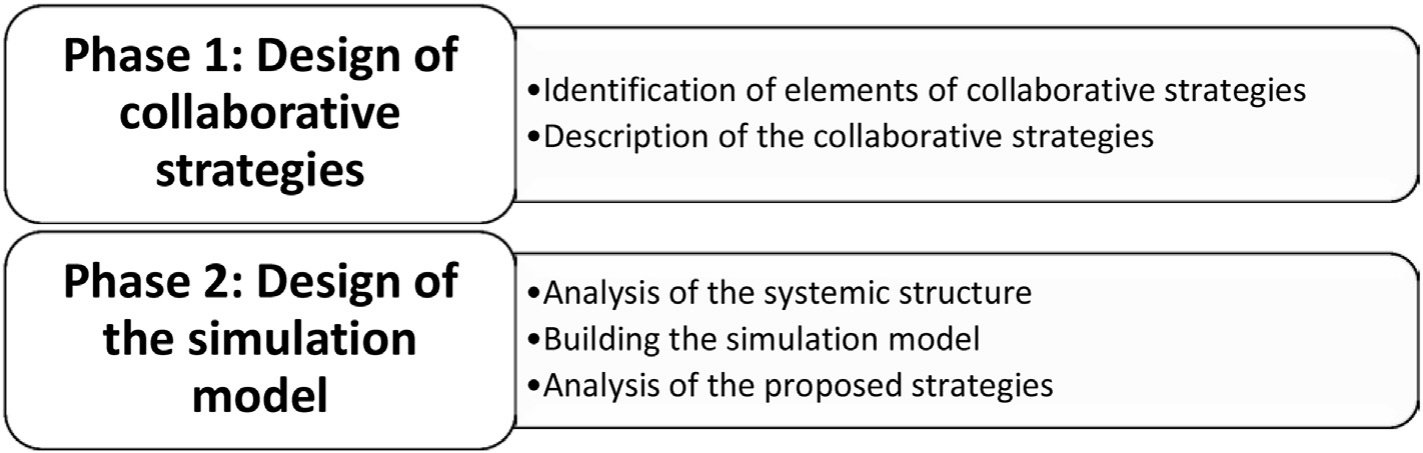
Proposed methodology composed by two phases.

The second phase corresponds to the design of the simulation model based on system dynamics. First, we present a conceptual model of the construction process of the simulation model, and we formulate the dynamic hypothesis for each proposed strategy. Then, we present the planning horizon, the stock and flow diagram of the simulation model, and the performance measures established to analyze the performance of the strategies proposed in the first phase. Thus, the simulation model is the tool to analyze the strategies and their impact on the average response times in different disaster situations. Figure 1 summarizes the proposed methodology. The following subsections present details about each phase of the methodology.

### 3.1 Design of collaborative strategies

Collaborative logistic strategies are achieved when two or more independent agencies work together (Simatupang and Sridharan, 2002) with the aim of providing an adequate response to the affected population in the right place, at the right time and in the amount required for managing disasters through the integration and exchange of resources and information (Richey et al., 2012). This definition corresponds to the response operations to sudden natural disasters of great magnitude, such as earthquakes, tsunamis, and floods (Van Wassenhove, 2006). The immediate response is performed during the first week after the events (Altay and Green, 2006). In this context, the autonomous entities correspond to: the affected local population; neighboring regions; local and national governments; foreign governments; NGOs; the UN; the private sector; donors; military forces; and multilateral, bilateral, and other specialized organizations (Oloruntoba and Gray, 2006). Further, the first ring donors are defined as the set of actors within proximity of the affected area of a disaster, that were not impacted by the disaster and thus can provide assistance in the first hours after the disaster. The first ring constitutes an important actor to be involved in relief operations. The resources covered by the definition are material, financial, and informational (Van Wassenhove, 2006); equipment and infrastructure are also included.

According to Cao and Zhang (2011) the term collaboration in supply chain can be described in function of seven components adaptable to humanitarian logistics: information exchange, congruence of objectives, synchronized decisions, aligned incentives, shared resources, team communication, and creation of joint knowledge. Given these components, the proposed strategies in this study are summarized as follows:

• Strategy 1: shared infrastructure without information exchange. This base strategy contemplates infrastructure shared between actors, without information exchange and represents the current operation of the system. The actors involved send available kits without knowing the quantities that have been sent by the other actor, increasing the inventory levels. To avoid using duplicity in the model, the infrastructure capacity does not been used is the capacity that the first ring donors will be able to use, and it also happens for the national and international actors who will use of the capacity not used neither by the local actor nor the first ring responders.
• Strategy 2: information exchange without shared infrastructure, where each actor has assigned its own percentage of infrastructure limiting the use of the corresponding area. In this strategy, the actors know the quantities of kit sent by each other actor, but they cannot know when to stop the send relief aids.
• Strategy 3: shared infrastructure and information exchange. In this strategy, the actors also know about quantities of kit sent by each other actor and they share infrastructure. Like strategy 1, it is necessary to avoid duplicity of capacity in the model.
• Strategy 4: shared infrastructure and information exchange about the delivered aids and variable to stop shipping. In addition to the shared use of infrastructure, this strategy implements the exchange of information about the missing quantities to be shipped and includes an order to stop the aid procurement process when the supplied kits are greater than the demand.

All the proposed strategies consider transportation modes (physical resources) as shared resources between the actors in the same level (local, first responders, and national, international and NGOs). Also, the four proposed strategies have the following common elements: more than three entities, foreign origin, public entities, non-governmental organizations, humanitarian aids of food and information as the shared resource. As base model or initial state, the subsystem of aid delivery incorporates only shared infrastructure (for example, airports, heliports, roads) and no exchange of information between actors. This configuration may cause material convergence and the relief needs will also be delayed by damage to infrastructure and facilities.

Thus, five assumptions are established for analysing and comparing the performance of the proposed strategies. These are:

• There are different geographical origins of the entities: These include local, national, and international entities. Military forces are considered as national agencies given that they commonly belong to the affected country. An exception would be the case of Haiti.
• There are different types of entities: Private, public (local, national, foreign, and military), NGOs, multilateral (including the UN) organizations, bilateral organizations, and donors.
• The shared resources could be: Material, financial, equipment, information, infrastructure, and staff.
• The supply chain flows are: Material, financial, information, and staff.
• The humanitarian aid assistance includes: Food (including water), health and sanitation, clothing, shelter, and non–food items.

### 3.2 Conceptual design of simulation model

Usually, humanitarian logistics systems are organized around the main needs of a humanitarian emergency, such as health, search, and rescue, aid delivery, among others. A clear example is the cluster approach of the United Nations (UN). Also, some countries have designed in a similar way national policies or national systems for risk reduction and disaster management.

**Figure 2:**
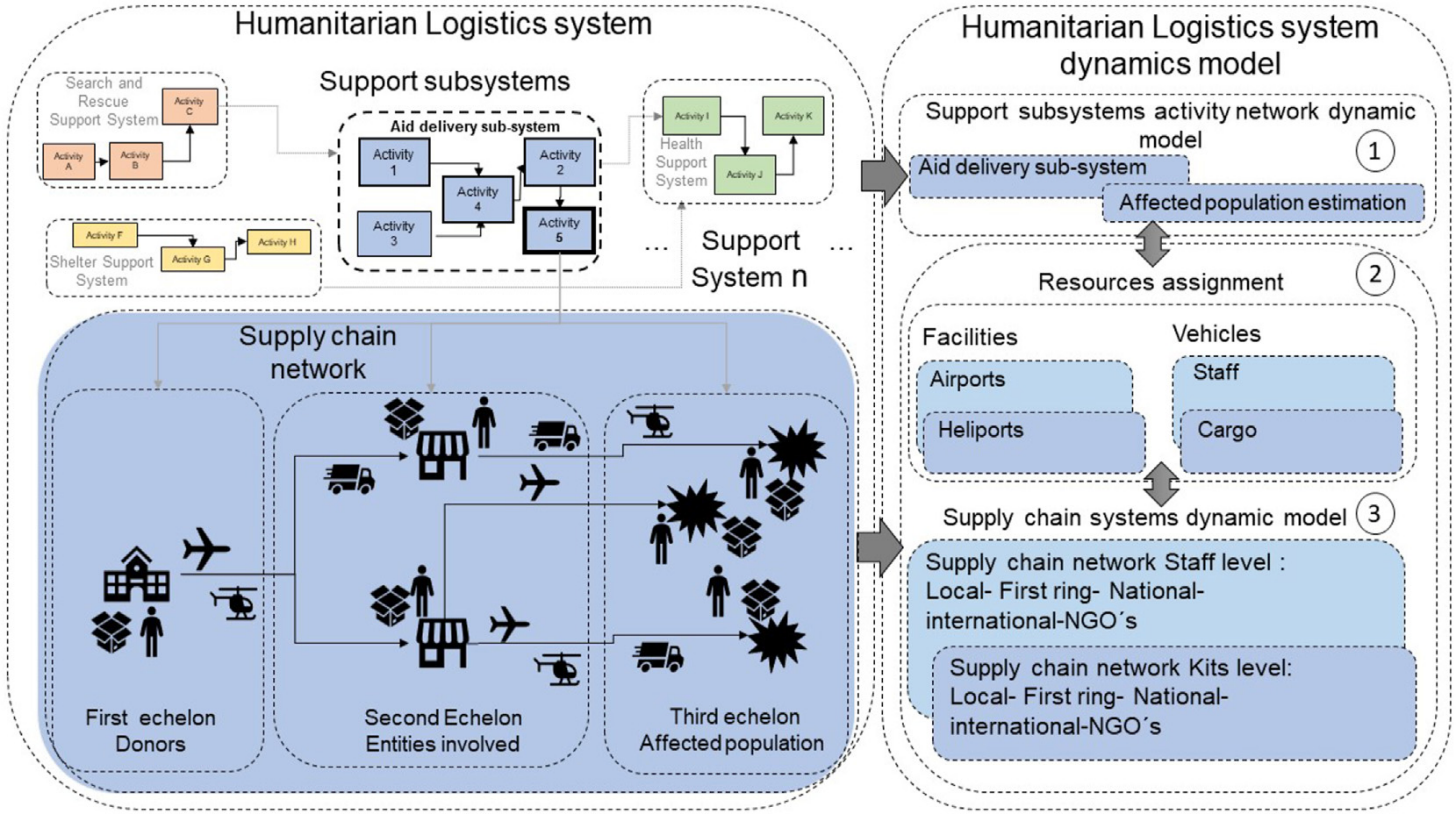
Conceptual model of the construction process of the simulation model.

In Figure 2, we present the conceptual model of the construction process for our simulation model composed by three sections: support subsystem model, resources assignment, and supply chain model.

The humanitarian logistics system is composed of different support subsystems. In this work, we are focused on the aid delivery sub-system in humanitarian operations. First, it is necessary to identify the main activities of each subsystem, precedence, and duration of each activity with the aim to construct the network activity model (module 1), adapting concepts of project management (Guzmán-Cortés et al., 2019).

In this case, once the disaster occurred, the aid delivery subsystem must perform five activities. These activities are: 1. requesting and/or confirming the aid requirements, 2. defining the aid procurement plan, 3. receiving the offer of national or international aid, 4. defining the amount of aid to deliver, and finally, 5. the last activity is the aid delivery. The aid delivery activity (number 5) is the most important from the point of view of logistics requirements, and it is linked with the estimation of the affected population which varies according to the disaster level.

Due to the importance of the aid delivery activity, the availability and allocation of resources are associated with this activity. Module 2 aims to assign the available resources considering vehicles to transport staff and cargo, and facilities such as airports and heliports.

Moreover, given that it is needed to estimate the duration of the aid delivery activity, a supply chain network is joined to this activity. Module 3 aims to model two supply chains associated with the flow of kits and staff, each one composed of three echelons.

The first echelon represents the donors that provide aids at different geographical regions. Local are in the same affected zone, regional or first ring are those located in the same metropolitan area of the affected city, national donors are in the same country where the disaster occurred, and international donor are from external countries. The second echelon is composed by the entities involves in the aid delivery. According to the level of the disaster and the amount of affected population, different actors are involved in the disaster response operations. For low disaster levels, the damage is the lowest, and local actors are responsible for managing the aid delivery. For disasters of medium level, the local and regional (first ring or metropolitan region) actors are involved. Finally, higher-level disasters require different actors to be involved: local, regional, and national and international actors (both are together).

Finally, the aid delivery process is also affected by the delays that can be caused by damage of infrastructure, availability of resources, long distances between the organizations and the affected population and the information flows between the different actors. Thus, the sub-system of aid delivery is subject to delays associated with first, the time required to call for aids to the different organizations and the staff call. These delays depend on the reaction time of the responding agency and normally take between 3 to 24 h (González Forero and González Rodríguez, 2013). The second delay is related to the traveling time and the number of trips that can be done by each vehicle, which depends on the distance, the infrastructure state, the staff levels, the amount of aid to be distributed and the capacity of vehicles, and the number of vehicles available, information obtained from public sources and fieldwork with stakeholders involved in the process. The last delay is associated with the capacity of aid delivery by the staff involved in the aid donation process, we assume as productivity factor per staff a quantity of six kits per hour.

### 3.3 Dynamic hypothesis

This section presents the dynamic hypothesis for each proposed strategy, which was explained previously in section 3.1. The fundamental causal-loop diagram is presented by (Guzmán Cortés et al., 2018).

**Figure 3:**
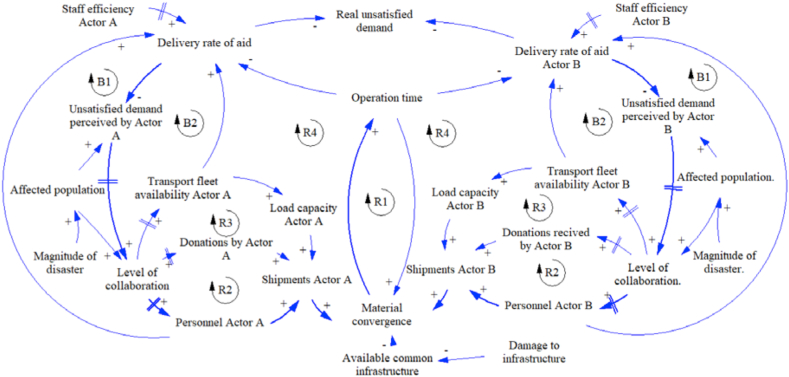
Dynamic hypothesis strategy 1.

For this work, the dynamic hypothesis of strategy 1 (base case) is presented in Figure 3. This strategy considers infrastructure shared without information exchange. In a general way, the figure shows the interaction between two actors as an example. The causal loop diagram presents four reinforcement cycles (R1, R2, R3, and R4) and two balance cycles (B1, B2) for each one.

According to the diagram, if the magnitude of the disaster increase, the affected population and the amount of unsatisfied demand perceived by each actor increase, too. This situation demands a higher level of collaboration represented in the type of actors and number of entities involved in the response. Then, if the level of collaboration increases, there would be more available resources such as fleet, donations, and staff, also increasing the shipment of aid done by each actor. The increase in shipments joined with the damage to the infrastructure generates material convergence and operations time increases, too. The higher the operation time, the lower rate of aid delivery. This rate is also affected by the capacity of the staff involved in the response. The shared infrastructure is represented by the variable named “available common infrastructure”. Due to the actors do not share information, they just send the aid according to the perceived demand for decreasing the real unsatisfied demand, but they cannot know about the amount of aid sent by each other.

**Figure 4:**
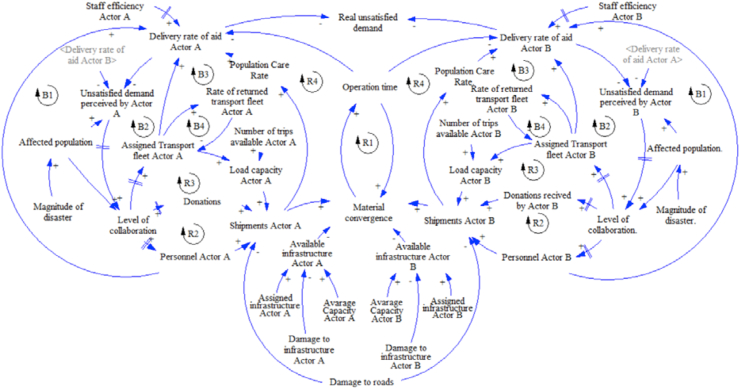
Dynamic hypothesis strategy 2.

The following causal loop diagrams for the additional proposed strategies have the same base structure from strategy 1 with some specific changes. The diagram of strategy 2 can be seen in Figure 4, with two differences. First, since the actors do not share infrastructure, each actor uses the own available infrastructure. And second, because the actors know the quantities of kit sent by each other actor, but they cannot know when to stop the send relief aids, this information exchange is represented with variable related to the rate of aid delivery, for example, actor A know the delivery rate of actor B. Like strategy 1, the actors send the aid according to the perceived demand for decreasing the real unsatisfied demand.

**Figure 5:**
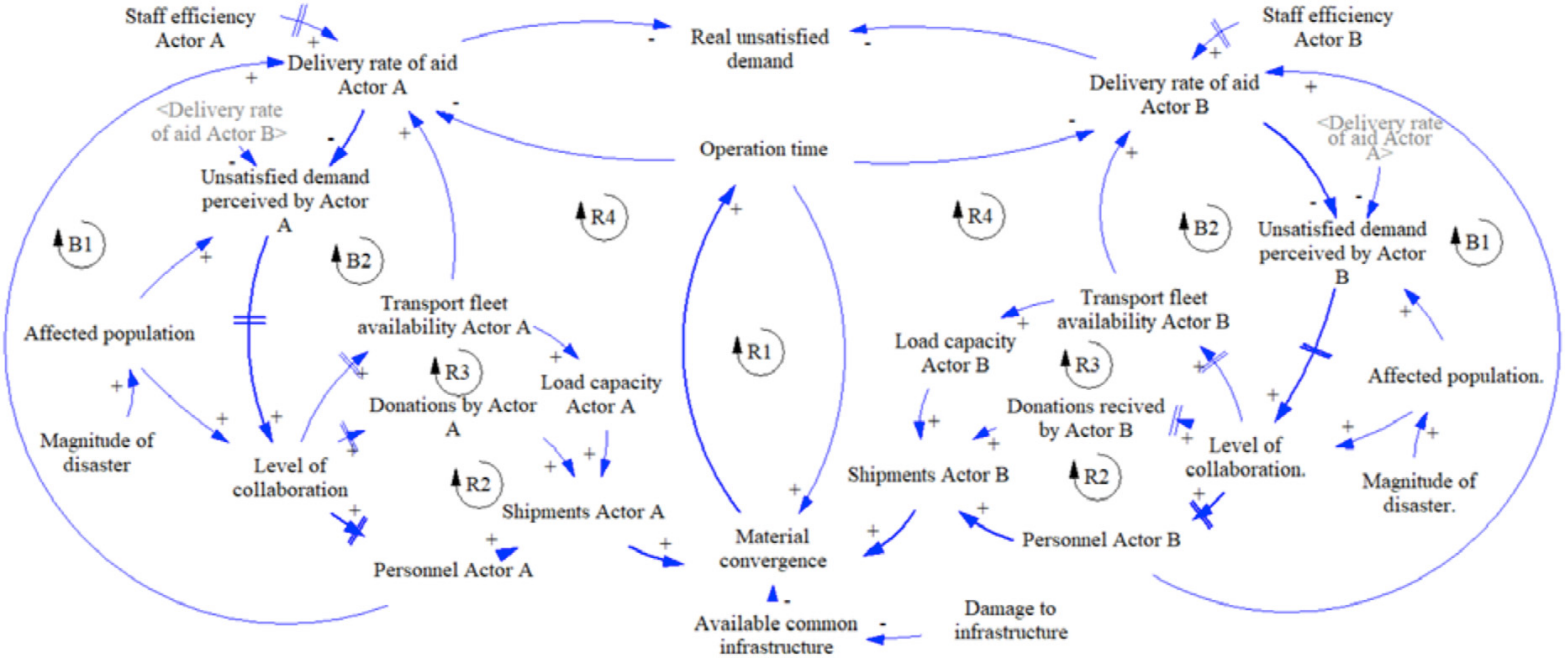
Dynamic hypothesis strategy 3.

Then, the causal loop diagram of strategy 3 is presented in Figure 5. In this strategy, the actors share infrastructure with an available common infrastructure, and they also exchange information about the quantities of kits send because they know the rate of delivery of each other.

**Figure 6:**
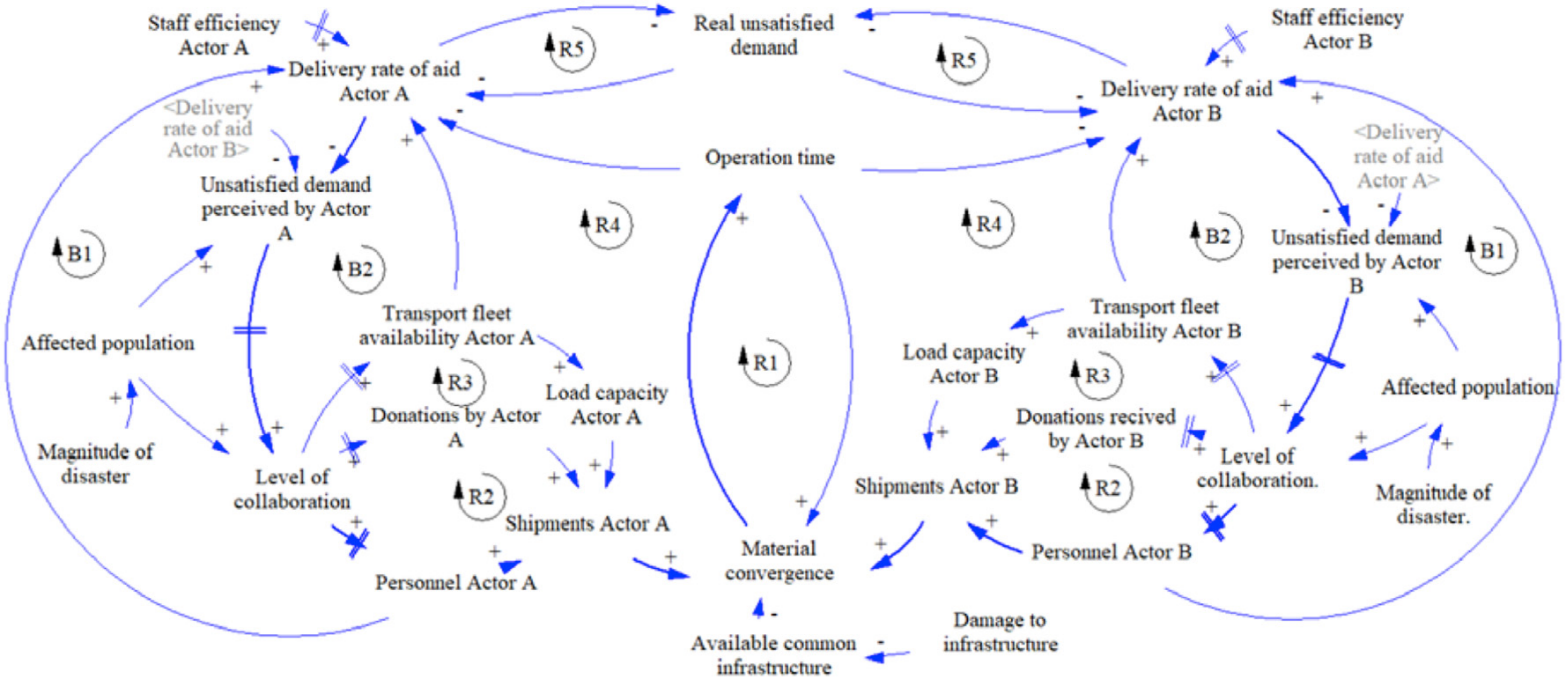
Dynamic hypothesis strategy 4.

Finally, the diagram for strategy 4 is shown in Figure 6. In this strategy, the actors share infrastructure, and they also exchange information about the quantities of kits sent by each actor and the missing quantities to satisfy the demand. It works as an order to stop the aid procurement process; this situation is represented by the new reinforcement cycle R5.

### 3.4 Planning horizon

The planning horizon for the simulation is defined at 168 h, with a time step of 1 h. Thus, we evaluate the first week after a disaster occurs. Nevertheless, it is expected that the aid delivery is completed within the first 72 h which are crucial to satisfied the needs of the affected people (Van Wassenhove, 2006).

### 3.5 Stock and flow diagram

The corresponding stock and flow diagram is explained according to the three sections of the proposed conceptual model. The details of each element in the stock and flow diagram proposed in this work are presented in Appendix 1. To sum up, the complete model of strategy 1 (base strategy) has 46 levels, 82 rates, and 179 auxiliaries/data/constants. To develop the simulation model, we have used the software Ventana Simulation Environment Vensim ® DSS for Windows Version 6.2.

**Figure 7:**
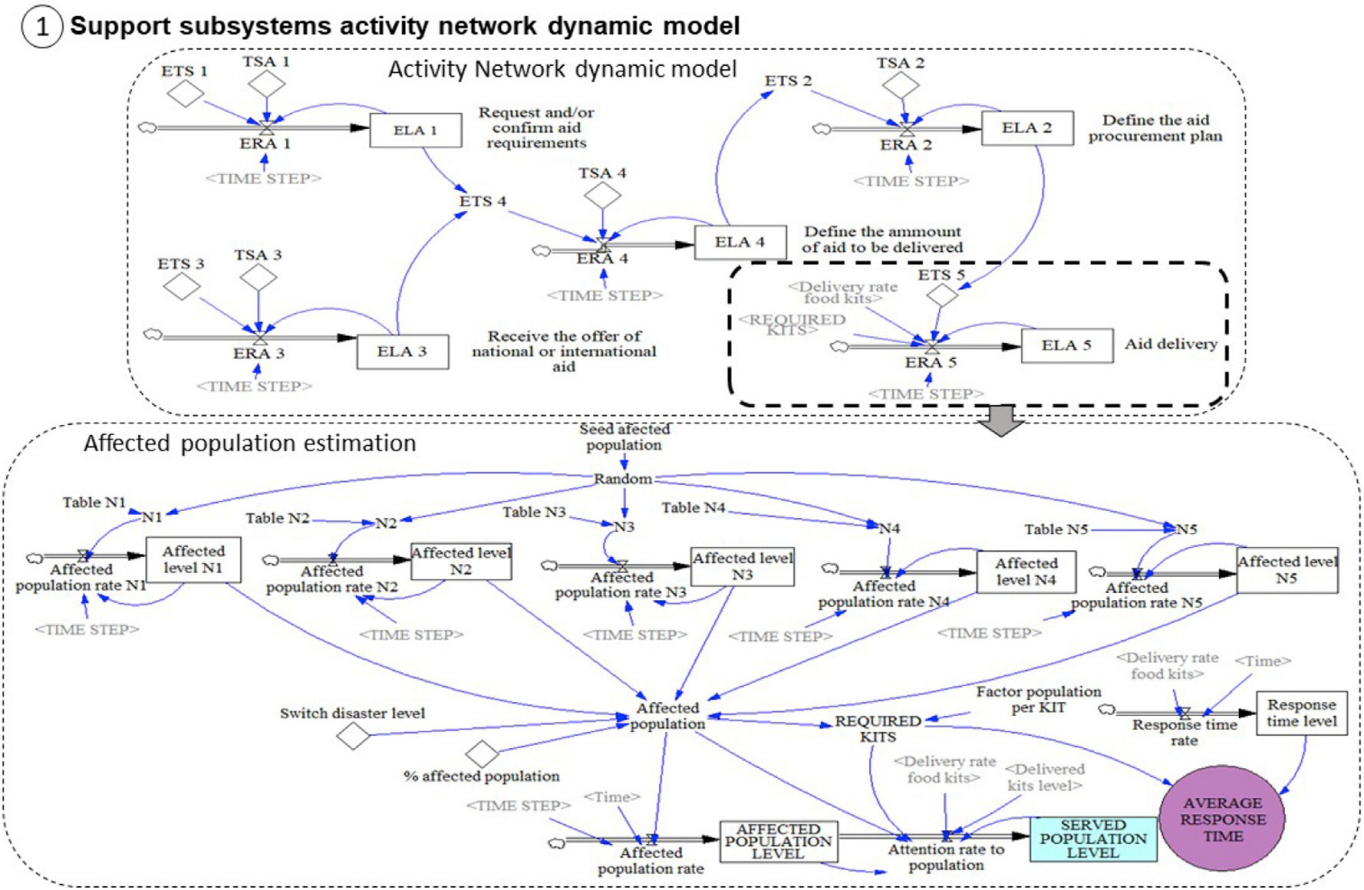
Stock and flow diagram section 1: activity network model and affected population generator.

The stock and flow diagram of section 1 of the conceptual model (support sub-system activity network model) is composed of two modules, and it is presented in Figure 7. The first module corresponds to the activity network model of the subsystem of aid delivery. This sub-system is composed of the five activities, as was mentioned previously.

This network is designed using activity on node (AON) representation. These activities are represented by execution levels of the activity (ELA) and each one of them has its own execution rate of the activity (ERA). All these activities are interrelated because of the precedence relationships. The precedence is indicated as information flows between the levels of previous activities and the execution of the technical standard (ETS) of the following activities. Finally, each activity has an associated technical standard (TSA) that corresponds to the expected duration of each activity. Given standard protocols of the activity network, in Table 1 the precedence constraints and expected duration of each activity is presented according to (González Forero and González Rodríguez, 2013). The expected duration of the Activity 5 depends on the performance of the two supply chains associated with the flows of material and staff in the three-echelon humanitarian supply chain presented previously.

**Table 1:** Description of the activity network of the subsystem.

CODE	NAME	EXPECTED DURATION (HOURS)	PRECEDENCE
**1**	Request and/or confirm the aid requirements	3	-
**3**	Receive the offer of the national or international aid	6	-
**4**	Define the amount of aid to be delivered	4	5.1, 5.3
**2**	Define the aid procurement plan	8	5.1, 5.3, 5.4
**5**	Aid delivery	Depends on the specific supply chain	5.1, 5.2, 5.3, 5.4,

The second module corresponds to the affected population generator. FOPAE & Alcaldía Mayor de Bogotá D.C (2008) propose a five-level disaster scale and depending on the disaster level the impact on the population varies. For low disaster levels (levels 1 and 2) the affected population is minimal. Level 3 presents a moderate impact, and for high levels (levels 4 and 5) there is significant impact.

**Figure 8:**
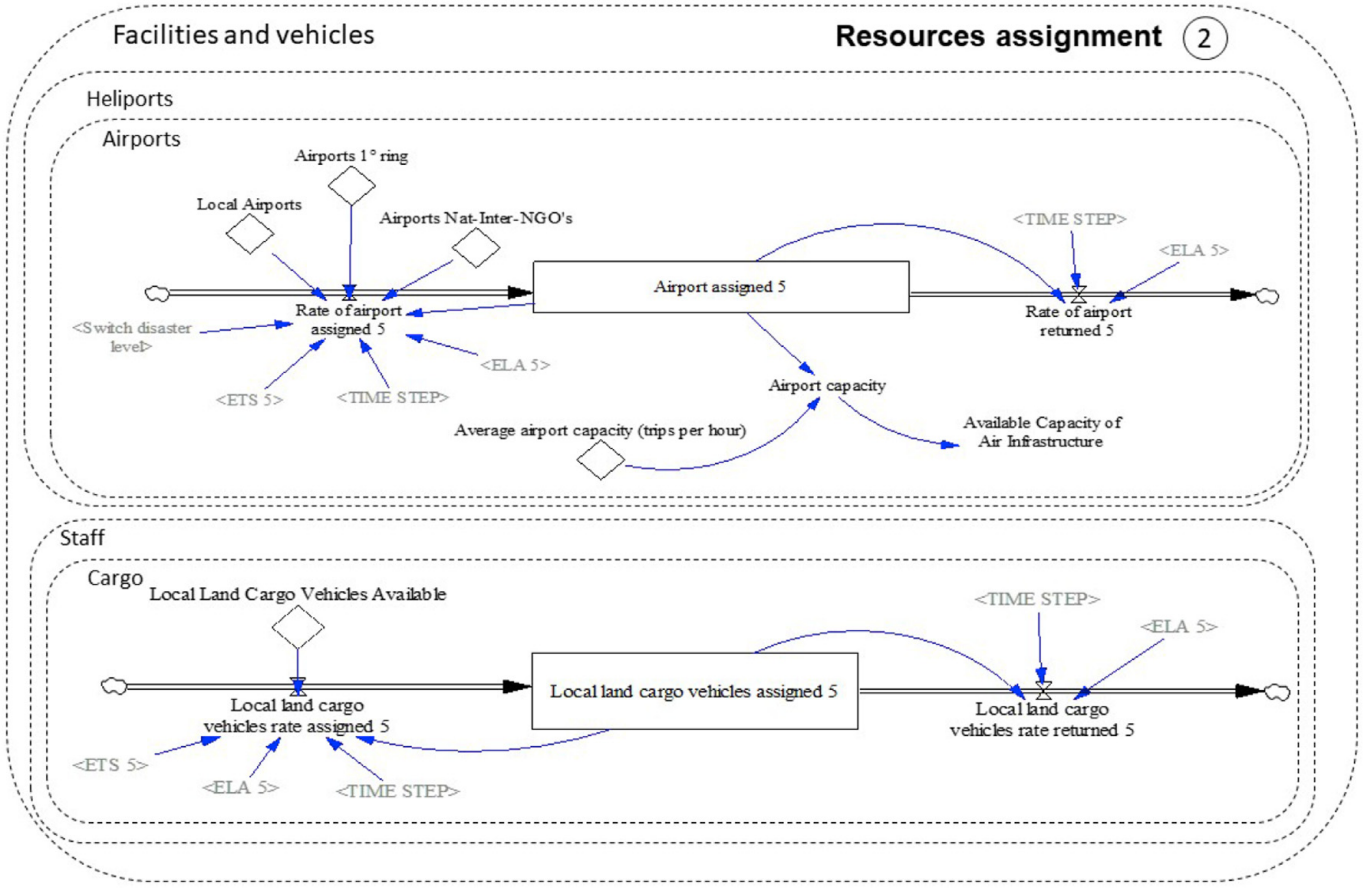
Stock and flow diagram section 2: resources assignment.

The stock and flow diagram representing section 2 of the conceptual model (resources assignment) can be seen in Figure 8. This figure presents the assignment process of cargo and staff to appropriate vehicles (land and air) and the assignment of infrastructure for air vehicles (airports and heliports). For the sake of brevity, Figure 8 presents an example of the assignment of infrastructure (airport) and an example of land cargo vehicle assignment. Related to land and air vehicles assignment, there are 12 similar diagrams (all with the same structure). Six diagrams are dedicated for assigning land and air cargo vehicles for each actor (local, regional - first ring, and national and international) and six to assign land and air vehicles for staff for each actor (local, regional-first ring, and national and international). The assignment of vehicles and staff are made when the activity of aid delivery (5) is activated.

Also, the assignments of vehicles are activated according to the disaster level. The local actor is always activated when a disaster occurs no matter the level of the disaster, regional or first ring actors are activated when the disaster level is 4, and national and international actors are activated when the disaster level is 5, joined with all the actors of the previous levels.

The assignment of physical resources of land and air vehicle for national, international, and NGO actors has the assumption that different entities can collaborate allowing to use a percentage of their own fleet to attend the emergency.

The assignment of airports and heliport is done in a similar way to the assignment of vehicles. The amounts of airports and heliports available for each actor are connected to the assignment rate, the infrastructure selection is made with the auxiliary variable "Switch Disaster Level", the assignment starts when the activities preceding activity 5 have ended and ends when the execution level of the activity is equal or greater than 100%.

The trips available per aircraft for each actor and the total number of trips per aircraft per hour are obtained from assigned air vehicles. Thus, the total trips available per aircraft correspond to the sum of the trips available for each actor. Similarly, it is carried out for both cargo and passenger aircraft.

**Figure 9:**
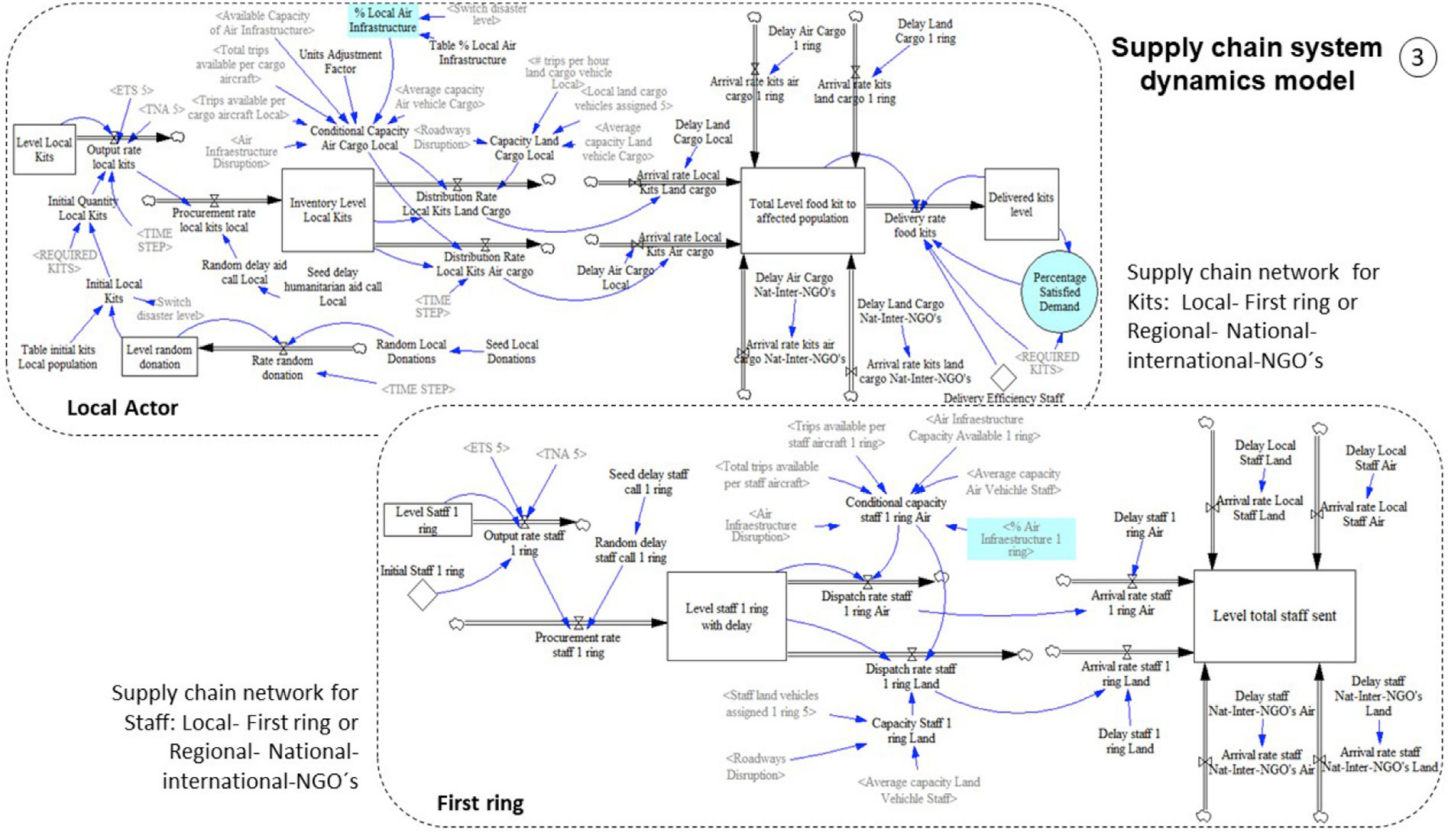
Stock and flow diagram section 3: supply chain system dynamics model.

The stock and flow diagram representing section 3 of the conceptual model (supply chain system dynamics model) is presented in Figure 9. In this view there are two examples of the two supply chains, the first one for the flow of kits considering the local actor, and the second one for the flow of staff to support the aid delivery, corresponding to the regional or first ring actor. The proposed supply chains were based on the general structure proposed by Peng et al. (2013), who present the Forrester diagram for the flow of materials and replenishment decisions in the post-disaster supply chain with an emphasis on inventory management.

Each supply chain proposed has three echelons. As it was explained before, the first one is constituted by donors, the second echelon is constituted by entities (local entities, first ring entities, and national, international, and NGOs entities) to support the affected area and finally, the third echelon corresponds to the affected population who receive the humanitarian aid.

In the example related to the local actor, the number of initial kits provided depends on the population not affected by the disaster. It is assumed that each unaffected person represents a potential food kit that could be donated. This amount is also a function of the disaster level and a randomized distribution that represents the real percentage of donations of the potential number of kits that could have been collected (between 0.1 and 0.5), and whose seed is represented by the auxiliary variable "Seed Local Donations". Additionally, the supply rate is affected by a delay in the call for humanitarian aid, which is represented by a random uniform distribution that varies between 3 and 24 h, whose seed is represented by the auxiliary variable "Seed delay humanitarian aid call Local".

Then, the humanitarian aid received from donors can be sent to the affected population by land and air, and the amount sent by each type of transport depends on the available capacities. Air capacity depends on the available capacity of the air infrastructure, the impact or disruption of the infrastructure according to the magnitude of the disaster, the average capacity of the aircraft, the number of trips available per cargo aircraft per hour, and the total trips available per cargo aircraft, variables that have already been described previously.

Likewise, the land capacity is obtained, which depends on the average capacity of the vehicles, the percentage of road disruption, the assigned land cargo vehicles, and the number of trips per hour that a vehicle can make. The percentages of disruption of air and land infrastructure were established through a look-up based on the disaster level.

Finally, the aid delivery process is carried out by the actors of the second echelon in the chain (entities involved). Each distribution or dispatching rate is connected to each arrival rates, which are affected by travel delays depending on the origin of the aid and the type of transportation mode.

It is necessary to highlight that for aid delivery to the affected population, both resources the kits (material flow) and the staff (personnel flow) must be available. The total quantity of staff sent can be found in a level in the staff supply chain which is linked by an information flow in the delivery rate of food kits. Additionally, the delivery capacity of kits per hour per each staff is considered. This delivery efficiency is represented in the model by the auxiliary variable "Delivery Efficiency Staff" and is assumed in 6 kits per hour per person. As can be seen in the example of Figure 9, the supply chain network for the staff has a similar structure to the supply chain for kits.

### 3.6 Performance measures

To analyze the results of the simulation model, the key performance indicators to be used are the average response time of kit delivery (ARTD), the delivery time of the first kit (DTFK) and the delivery time delivery of the last kit (DTLK). Table 2 presents the experimental design where each strategy is tested for each level of disaster.

**Table 2:** Experimental design.

	LEVEL OF DISASTER				
**Strategies**	**Level 1**	**Level 2**	**Level 3**	**Level 4**	**Level 5**
**Strategy 1**	ARTD	ARTD	ARTD	ARTD	ARTD
	TEFK	TEFK	TEFK	TEFK	TEFK
	TELK	TELK	TELK	TELK	TELK
**Strategy 2**	ARTD	ARTD	ARTD	ARTD	ARTD
	TEFK	TEFK	TEFK	TEFK	TEFK
	TELK	TELK	TELK	TELK	TELK
**Strategy 3**	ARTD	ARTD	ARTD	ARTD	ARTD
	TEFK	TEFK	TEFK	TEFK	TEFK
	TELK	TELK	TELK	TELK	TELK
**Strategy 4**	ARTD	ARTD	ARTD	ARTD	ARTD
	TEFK	TEFK	TEFK	TEFK	TEFK
	TELK	TELK	TELK	TELK	TELK

To determine the number of replications for each strategy, the Eq. (1) is used with an estimated error of 5%:

(1) $$n=\frac{S^{2}*{Z_{\alpha /2}}^{2}}{e^{2}}$$

Given that in Eq. (1) the standard deviation is not known, 10 preliminary runs are made for each strategy and each level. Once it is known the standard deviation, the number of replications is calculated by Eq. (1). Then, to find significative differences between the strategies, the results are compared using the key performance indicators with the parametric tests Tukey and Duncan.

## 4 Case study

This section presents the subsystem of humanitarian aid delivery in Colombia to present the case that motivated this study. In this context, the processes performed by the national agencies related to humanitarian aid distribution, logistic support (capabilities and resources), and responsibilities are characterized. This characterization is obtained through the analysis of primary sources such as: official documents, protocols, and information obtained with interviews with staff involved in the decision making of the organizations. First, we present the seismic risk scenarios of the case study. Then, we explain the actors that must be coordinated in the disaster relief operations in the case with the proposed strategies. Finally, we detail the available resources and their capabilities for humanitarian aid distribution in the risk scenarios.

### 4.1 Disaster scenarios in the city of bogota

The city of Bogota-Colombia is chosen as case study since it has an estimated population of more than 8 million people, with an area of 384 square kilometers and it is one of the cities with highest vulnerability to natural disasters in South America (Riaño et al., 2021) and high population density (more than 24,000 inhabitants per square km). The city is located in the central region of Colombia, about 1000km north the Ecuadorian line, in a 2600 m above sea level plateau in the Andean Eastern Cordillera. Further, it is located on a lacustrine soil deposit surrounded by hills that consists of mainly soft deposits up to 500 m deep, which constitutes a potential risk for a catastrophic earthquake controlled by the Eastern Frontal fault system (Riaño et al., 2021).

Our study considers five different level of disaster following the categories established by (FOPAE & Alcaldía Mayor de Bogotá D.C, 2008) where they determined five levels depending on the magnitude and complexity of the emergency, and the capacity of response. Each scenario has different magnitude of the disaster. The number of potential affected population in Bogotá is estimated using the Seismic Damage Index in case of an earthquake with a magnitude of 7.0 on the Richter scale in Bogotá (Fondo de Prevención y Atención de Emergencias-FOPAE, 2011). Table 3 depicts the estimation of ranges of the number of the affected population per level of the disaster:

**Table 3:** Ranges of estimated affected population in function of the level of disaster.

Level of disaster	Lower bound of affected population	Upper bound of affected population
**1**	0	163727
**2**	163727	810239
**3**	810239	1935114
**4**	1935114	2909080
**5**	2909080	4603970

In these scenarios, the first ring of the city of Bogotá includes actors located in six municipalities (named “La Calera", “Chía”, “Cota”, “Funza”, “Mosquera”, and “Soacha”). These are characterized by being municipalities with significant population flow and commercial trade with the city of Bogota. These municipalities account for 32% of the state population (“Cundinamarca”) and its territorial extension is almost 800 km^2^ (Alcaldía Mayor de Bogotá D.C, 2010).

### 4.2 Involved actors and responsibilities in the case study

In Colombia, humanitarian operations are coordinated by the National Unit for the Management of Risk of Disasters (*Unidad Nacional Para La Gestión Del Riesgo De Desastres - UNGRD* in Spanish) and the National System for Disaster Risk Management (*Sistema Nacional de Gestión del Riesgo de Desastres - SNGRD* in Spanish). This system assigns key logistic activities to supporting subsystems. These activities are accessibility and transportation, healthcare, search and rescue, firefighting, handle of hazardous materials, assisted evacuation, humanitarian aid delivery, shelter, water, basic services, sanitation and hygiene, debris management and reconstruction, telecommunications, among others. Each one of these subsystems has assigned a responsible agency to coordinate the process with the assistance of the required agencies. Thus, in this case there is multiplicity of agents and objectives in the humanitarian operations and, therefore, the response times are affected (Tatham and Houghton, 2011). Given that during the disaster, the challenge is to organize and coordinate the logistic processes needed to deliver aid in the affected regions, it is necessary to create alliances between the involved agencies, donors or institutions that can provide support to improve the response times of the sub system of aid delivery.

The UNGRD has three committees. These are the knowledge of risk committee, the risk reduction committee, and disaster management committee (Decreto 4147 de 2011, 2011). The last committee coordinates the aid management subsystem. This subsystem oversees the immediate response in case of a disaster by analyzing the aid requirement, managing physical and human resources required for the aid distribution, request for national or international aids, and organizing and coordinating the logistics for the reception, delivery, and administration of aid to the affected regions.

In terms of the agents involved in the aid distribution process (Oloruntoba and Gray, 2006), identify six agents in the supply chain: donor governments, international agencies, non-governmental international agencies, non-governmental local agencies, local partners and finally the aid consumers or beneficiaries (affected population). In the case of the Colombian UNGRD, the actors involved in the aid distribution can be defined as: Colombian Red Cross that evaluate the damages and needs, distribute food and non-food assistance, and manage temporary shelters. The second agent involved is the Colombian Civil Defense which executes specific projects or programs for disaster prevention. Also, the Colombian Ministry of Foreign Affairs coordinates and formulates the international cooperation policy for disaster management. Further, the Colombian Ministry of Health and Social Protection manages the aid distributions related to healthcare requirements. The Colombian National Agency of Taxes and Customs oversees the importation process of services and goods. The Colombian National Police evaluates the damages, supports the aid distribution to the affected population and guarantees the public order. Finally, the Colombian National Fund for Disaster Risk Management manages the financial resources for the implementation and execution of actions and policies for disaster risk management.

Thus, for different levels of the disaster, the response protocols are different. That is, for disasters with lower levels of damage, only local agencies (governmental and non-governmental) are involved in the response. As the level of damage of the disaster increases, more agencies are involved in the response, which include national and international agencies.

### 4.3 Resources and capabilities for humanitarian aid distribution in the case study

Three types of resources controlled by the agencies are considered in the proposed simulation model since these are directly involved in the management and delivery of aid. These are Staff (operators and volunteers), vehicles to transport cargo and staff, and facilities where the cross-docking, warehousing, and other logistic operations take place.

Also, in the case of a disaster in the city of Bogotá, the following considerations are explicitly modeled in our case study: First, information about the access roads to Bogotá allow us to determine the transportation time to make the aid delivery in the city. Second, the number of available staff for the disaster response in the different response agencies we estimate the delivery rate to the affected population. Third, Physical resources available such as ambulances, vehicles (trucks, campers, lift trucks, helicopters among others) that allow us to estimate the capacity of preparation and distribution of aid kits.

The data for this case study is collected from primary and secondary sources. Thus, interviews to the agencies involved in the aid distribution are made. Also, we performed an analysis of official documents related to operational plans and strategies. Thus, to analyze the collaboration strategies of the first ring of influence to the city of study, we consider the dynamics of the information and resource sharing processes between the actors at neighbor municipalities and the affected area.

The average capacity to transport staff and cargo to the affected city for the air transportation mode is estimated by a weighted average considering three major aircraft operators, the Colombian government-owned airline, the largest private commercial airline, and the Colombian air force. For example, the estimated average capacity to transport staff considering the type of airplanes, the fleet sizes, the number of seats is presented in Table 4, Table 5, and Table 6. Then, into the model, it is considered that a random percentage up to 30% represents the maximum fleet available by each actor for the emergency response.

**Table 4:** Capacity of The Colombian government-owned airline for transportation of staff.

Airplane	Seats	Quantity	Total
Embraer ERJ 170-100LR	76	1	13
Embraer ERJ 145LR	50	2	
Dornier Do 328-100	32	4	
ATR 42-500	46	4	
ATR 72–212A	70	2	
Average Capacity (Passengers)	54,8

**Table 5:** Capacity available for staff transportation of a private commercial airline. Source (Avianca, 2014).

Airplane	Seats	Quantity	Total
Airbus A321	194	5	101
Airbus A320-200	150	54	
Airbus A319-100	120	25	
Airbus A318-100	100	10	
ATR-72	68	7	
Average Capacity (Passengers)	126,4

**Table 6:** Capacity available for staff transportation of Air Force. Source (Flightglobal Insight and RUAG Aviation, 2013).

Airplane	Model	Quantity in service	Capacity (Passengers)
Bell 205		2	14
Bell 212 Twin Huey		10	15
Bell UH-1	UH-1H	20	14
McDonnell Douglas MD 500 Defender	369HM/MD 530FF	4	5

Bogotá city has two airports (El Dorado airport and Guaymaral airport) and 40 heliports, including both public and private. The first ring has 2 available heliports available for humanitarian agencies and NGOs. As for airport capacity, El Dorado airport has an arrival rate 30 flights per hour (Unidad de gestión de afluencia del tránsito aéreo, 2014). In the case of heliports, the average capacity of was unrestricted given the possibility that helicopters have to land at alternative locations such as parks and open fields. To calculate the total number of trips available per aircraft, it is necessary to establish the expected time for loading, unloading, travelling and potential delays per aircraft. This data presented in Table 7.

**Table 7:** Number of trips for aircraft of each actor.

Origin of transportation operation	Load and Unload Time [h]	Flight Time [h]	Delay [h]	Number of trips
**Local**	0,33	0,11	0,55	1,82
**1° ring**	0,33	0,25	0,83	1,20
**Nat, Inter- NGO**	0,33	6,15	12,63	0,08

Loading and unloading an aircraft with parallel warehouses takes approximately 20 min. The average flight time of an aircraft in Bogotá is 7 min (0.11 h) according to statistics from helicopter taxi services. The average flight time between the first ring and Bogotá is approximately 15 min. Also, the average travel time for a cargo originated from a national or international NGO is 6.15 h. We assumed that the cargo could come from the largest commercial destinations in the world.

As for local transportation in Bogota, according to Cámara de Comercio de Bogotá and Universidad de los Andes (2011), the average time of a trip inside the city of Bogotá is 0.88876 h. For the first ring, the average travel times correspond to the estimation made by the mobility observatory (Cámara de Comercio de Bogota & Universidad de los Andes, 2007) which is 0.9774 h.

In the case of kits that come from different parts of the country, the distances from Bogotá to the main cities of the country were taken, the average speed of a cargo vehicle on the road national is 35 km per hour (Proexport Colombia, 2012) and the average travel time by land vehicle was estimated in a value of 18.3378 h. In this way, with the average travel times, it was possible to obtain the delays for the delivery of kits and the number of trips per hour of a land cargo vehicle.

The initial number of staff for the local actor and the first ring of influence is shown in Table 8 and were obtained because of the characterization of the aid subsystem. The initial number of staff for the third actor (national, international, and NGOs) is defined as a random number between 0 and 200000 volunteers and it is assumed that only a percentage of this number could reach the affected area within the first hours after a disaster occurs. This staff would join volunteer NGO staff.

**Table 8:** Number of staff for local actor and first ring of metropolitan influence.

Local Staff		
Operative		11357
Volunteer		37020
Total		48377
Staff 1° ring		
Operative	885	
Volunteer	1409	
Total	2294	

This case study is noteworthy since the city of Bogota is one of the largest cities in Latin America, with significant risk of a catastrophic earthquake, and can provide insights which can be appropriate to other large cities in the world under similar conditions. Comparing the impact of the different collaboration strategies for disaster relief can be the groundwork for designing public policy to design better prepared and more resilient cities.

## 5 Results and analysis

In this section, we will present the results for the model validation process, and the comparative results of each proposed strategy to provide managerial insights about the collaborative planning process for aid distribution in different disaster relief scenarios.

### 5.1 Validation

The consistency of the model was verified to get the desired behaviors. The behaviors evaluated were:

• The aid delivered cannot be greater than the donations available.
• The aid sent cannot be greater than the inventory available.
• The capacity of each transportation mode must be respected to avoid negative inventory levels of kits.
• Aid delivery consistent with the staff available and their efficiency.
• The staff sent cannot be greater than the staff available.
• The capacity of each transportation mode for staff must be respected.
• The number of kits required should not be exceeded according to the affected population.

Additionally, two tests of extreme conditions were performed to validate the model.

Test 1. Available Vehicles (Value 0): if the subsystem does not have vehicles available to transport and distribute the kits, there must be no assigned vehicles, the aid cannot be sent, there must be no kits delivered and therefore the percentage of satisfied demand is zero. This case is performed for disaster level 1 in which only the local actor intervenes.

Test 2. Donations (Value 0): if there are no aid donations (considering that is a food kit that cannot be pre-positioned), the inventory level of kits and distribution rates are zero. Therefore, the level of kits delivered, and the percentage of satisfied demand are also zero. This test was also carried out for disaster level 1.

### 5.2 Results

The model presented in Section 4.4 is simulated over a planning horizon of seven days (168 h) that corresponds to the expected maximum response time according to the Colombian protocols for aid distribution.

According to Section 3.6, the key performance indicators for each strategy and level of disaster are the average time of kit delivery (ARTD), the delivery time of the first kit (DTFK), and the delivery time delivery of the last kit (DTLK). Following the protocol, 10 preliminary runs were made for each strategy and each level and using Eq. (1) were obtained 28 runs for the first three strategies and 40 for the last one.

**Figure 10:**
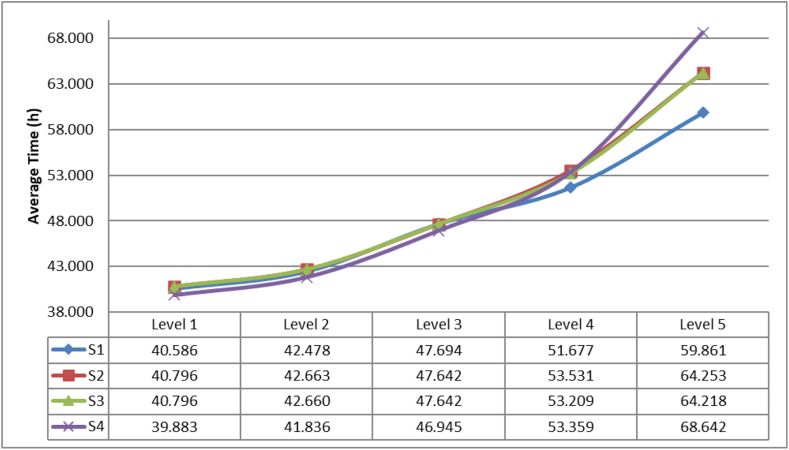
Average response time of aid delivery.

The first result corresponds to the average response time of kit delivery (ARTD) which is presented in Figure 10.

In Figure 10 it can be concluded that the strategy 4 presents the best performances for disasters with level 1, 2 or 3. For the levels 4 and 5 the best results are obtained by the strategy 1. The statistical results of the parametric test Tukey and Duncan allow us to conclude that only exist a significative difference between the strategies 1 and 4 with a p value of 0.024 and is highly observable in the level 5.

**Figure 11:**
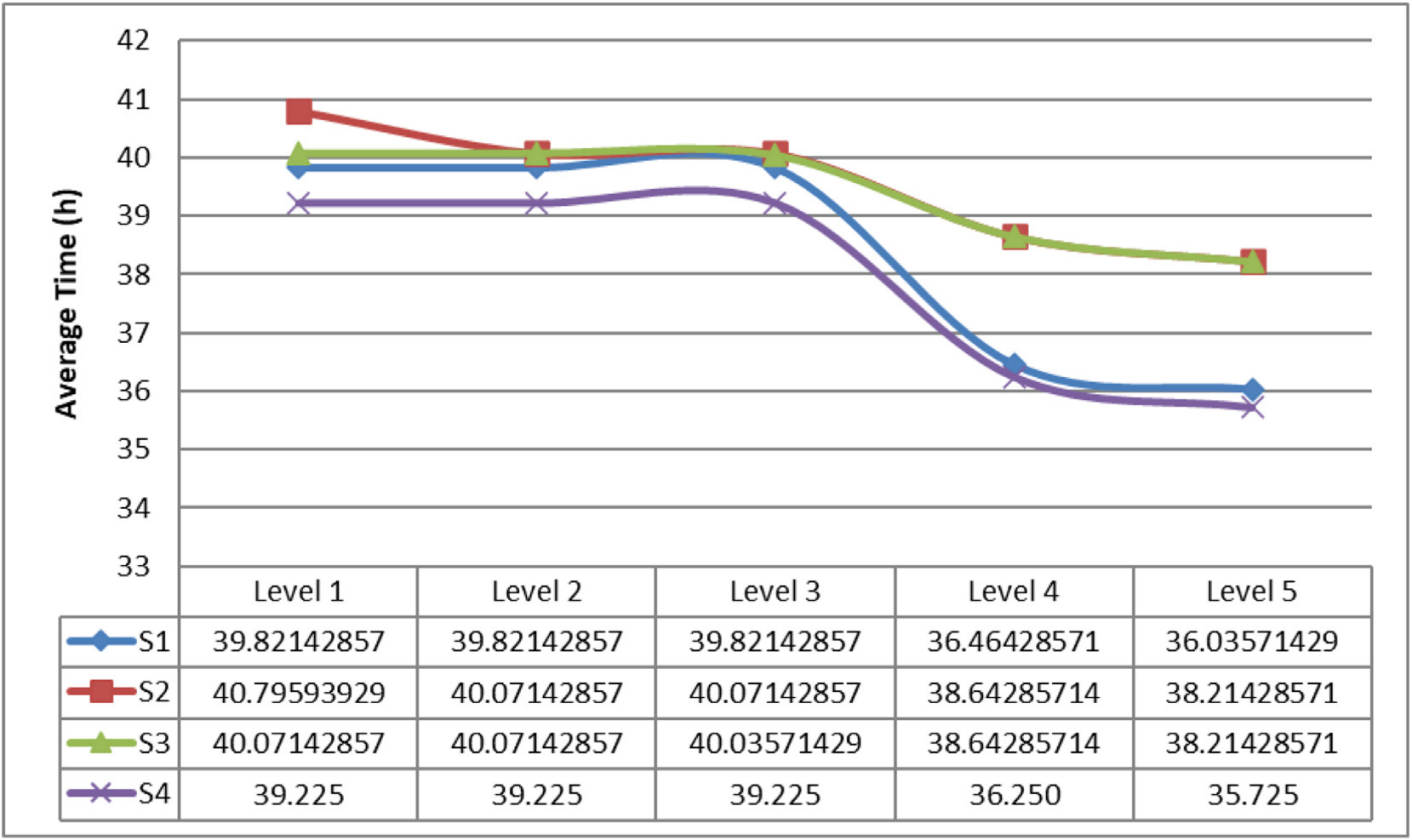
Average time of delivery for the first kit.

A second comparison is made with the average time of delivery for the first kit (DTFK). These results are presented in Figure 11.

It can be concluded that strategy 4 overperform the rest of the strategies for the 5 different levels of disasters. Analyzing the statistical test there are significantly differences between the strategies 2 and 4 and between the strategies 3 and 4.

**Figure 12:**
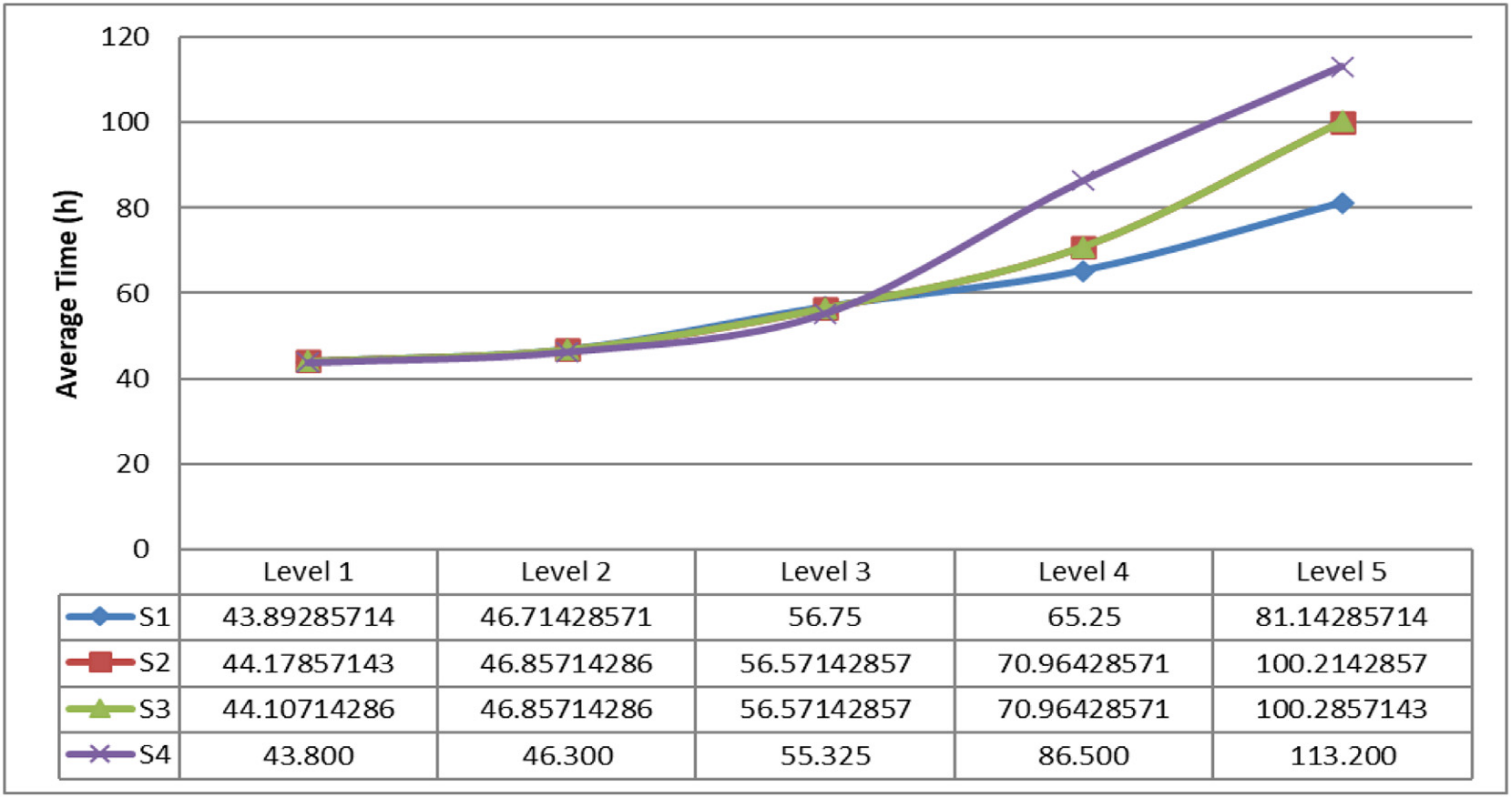
Average delivery time for the last kit.

Finally, the average times of delivery for the last kit (DTLK) are presented in Figure 12. It can be concluded that for the level 1, 2 and 3 the results are close, but for level 4 and 5 the best performance is obtained by the strategy 1. Nevertheless, the statistical test shows that there is statistical difference between the strategies 1 and 2, 1 and 3, 1 and 4, 2 and 4 and 3 and 4.

Given the results presented above, it can be concluded that strategies 2 and 3 for the level of disaster 1, 2 and 3 there is not a direct impact of sharing infrastructure because there is enough capacity available to support the distribution to the affected population. When in some disaster levels is required the regional/local or international cooperation, strategies 2 and 3 obtain higher response times compared with the strategy 1 which can be interpreted because of the high amount of affected population that can be easily supported if all the actors deliver kits in a parallel way.

If the level of disaster is 1, 2 or 3 it is desirable to use the strategy 4 and when there is an increasing of the affected population (level 5 of disaster), the response times also increases because the different kits delivered by the different organizations must wait for distribute the aid given the capacity of the infrastructure.

To choose and adequate strategy is mandatory not only to consider the average times but also consider the monetary resources necessary for support the affected population. Another important element to consider corresponds to the life cycle of the kits that on average are between 8 to 10 days. In this sense, is necessary to define if it is cheaper to keep kits on inventory or not.

**Figure 13:**
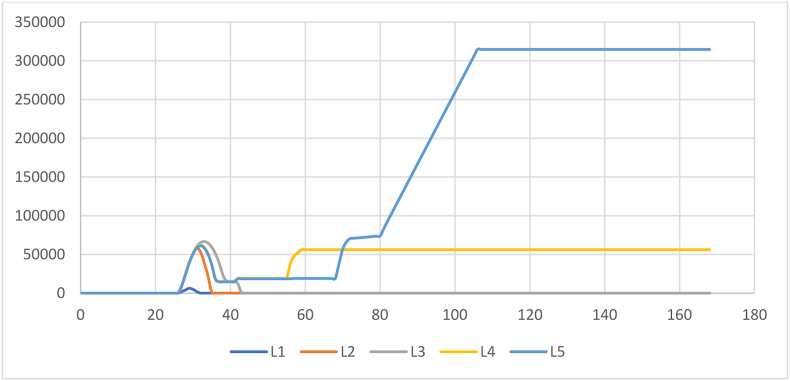
Inventory level by level of disaster in strategy 1.

As can be seen in the above Figure 10, the lower average response times for level of disaster 4 y 5 were obtained with strategy 1. However, it is also important to analyze the behavior of the inventory level in this strategy for each level of disaster. According to the results presented in Figure 13, the higher level of disaster the higher level of inventory, generating congestion in the system. Although the demand is satisfied totally, the accumulation of inventory could increase holding and operational costs.

Given this, with the main objective of avoid the accumulation of aid kits as it is presented in the strategy 1, also is desirable to reduce the average response times of the strategy 4 which have the higher values for levels 4 and 5. Therefore, a new strategy (strategy 5) is proposed with the aim to improve the strategies 1 and 4.

**Figure 14:**
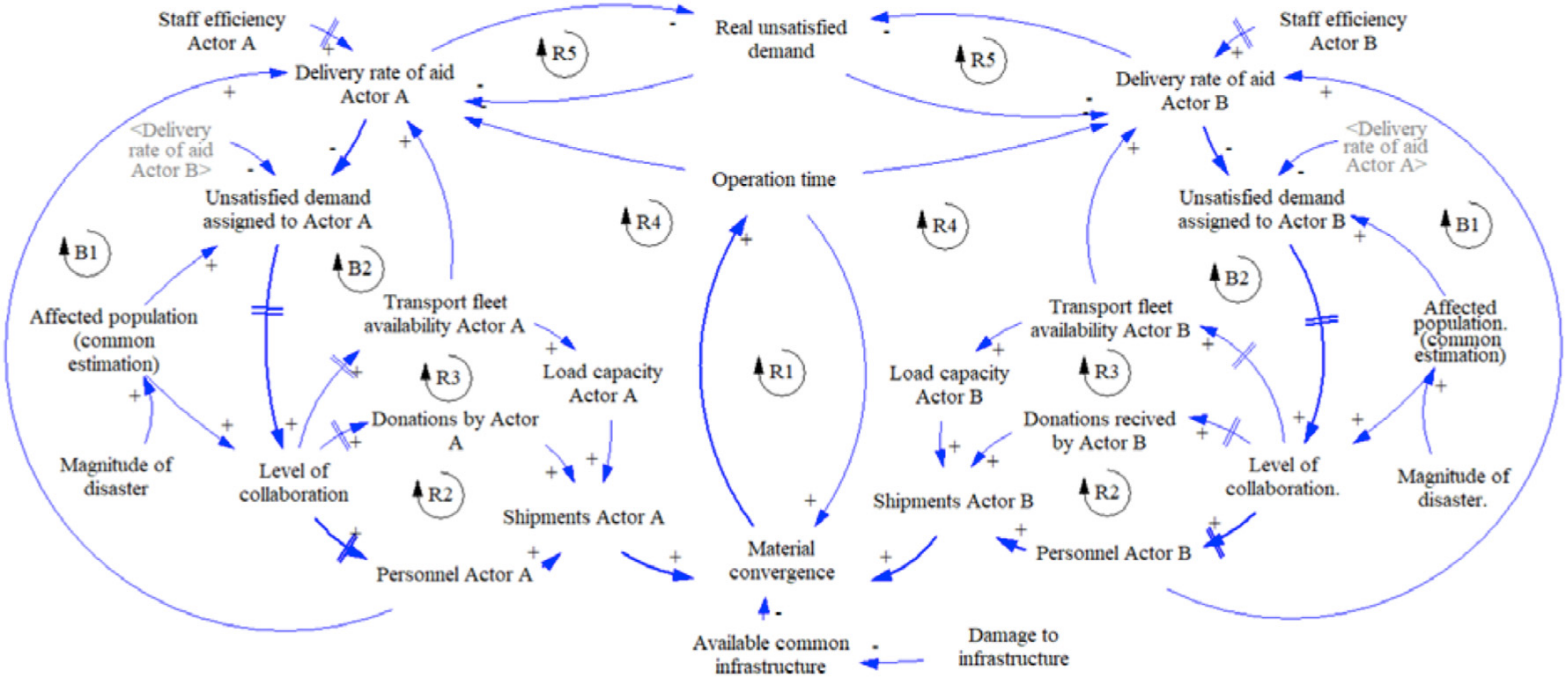
Dynamic hypothesis strategy 5.

Strategy 5 considers shared infrastructure and information exchange about the amount of aid necessary with creation of joint knowledge. This strategy incorporates a mathematical model to define the optimal shipping policies with the objective of minimizing the total travel time by the different transportations modes. The modified causal loop diagram of this strategy can be seen in Figure 14. The main difference is the actor has to satisfy the assigned demand instead of the perceived demand, given that they make decisions together using the mathematical model.

The following is the elements of the mathematical model used:

Index:

i=typeofcargotransportationmode(1=airplane,2=land)

j=typeofactorinvolved(1=local,2=1°ring,3=national,internationalandONGs)

Parameters:

${Cap}_{ij}=Kits\;capacity\;of\;the\;transportation\;mode\;i\;for\;actor\;j\;per\;hour$

QR=Ammountofkitsrequiredforthelevelofdissaster

${TV}_{ij}=$ *Travel time in hours or delay in the delivery of kits in the trasportation mode i for actor j*

Variables:

$H_{ij}=$ *Ammount of travel time used by each transportation mode i for each actor j*

${HT}_{ij}=$ *Ammount of travel time used by each transportation mode i for each actor j*

${PE}_{ij}=Delivery\;policy\;for\;each\;transportation\;mode\;i\;for\;each\;actor\;j$

The following is the mathematical model used:

(2) $$FO=\mathrm{MIN}\left\{\frac{\arg\;\max\;\left({HT}_{ij}\right)}{i,j}\right\}$$

Subject to:

(3) $$\sum_{i}\sum_{j}({\;Cap}_{ij}*H_{ij})\geq QR$$

(4) $${PE}_{ij}={Cap}_{ij}*H_{ij}\forall i,j\;$$

(5) $${HT}_{ij}=H_{ij}+if\;\left(H_{ij}>0,then\;{TV}_{ij}\;else\;0\right)\forall i,j\;$$

(6) $${PE}_{ij},{HT}_{ij},H_{ij}\geq 0\forall i,j\;$$

**Figure 15:**
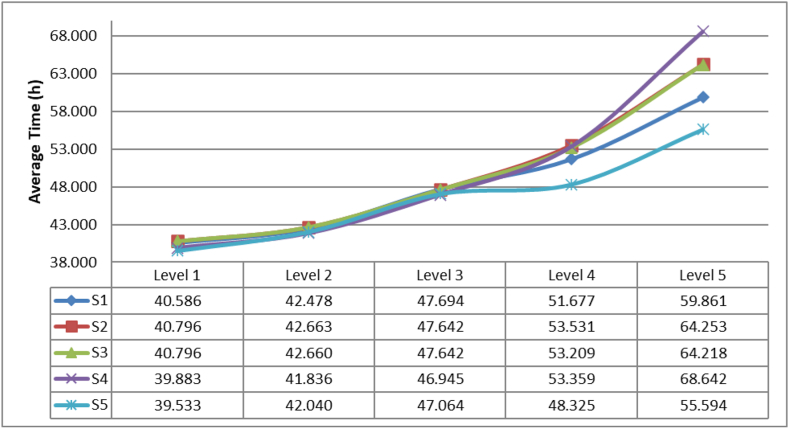
Average response time of aid delivery with the new proposed strategy (S5).

The objective function detailed in Eq. (2) represents the accumulation of the delivery times. Eq. (3) define the demand satisfaction given the amount of kits required. In Eq. (4) the delivery policy is defined by considering the capacity of transportation in hours and the amount of travel time used. In Eq. (5) the non-linear constraint considers the accumulation of the travel time and delays by each type of transportation time and each actor. Finally, Eq. (6) represent the type of variables. The solution of the mathematical model is used as an input of the simulation model integrating the amount of delivery rates. With this new information 10 more runs were performed to obtain the same metrics analyzed and analyze the inventory levels and compare this new strategy with the first four analyzed before. These results are presented in Figure 15.

It can be observed that the average response times of the subsystem are improved even with lower values that those obtained by the strategy 1. Therefore, it can be concluded that the strategy proposed presents the best results over the simulations and hence it can be chosen as the best policy to reduce the response times of the subsystem and distribute all the aid kits required to the overall affected population, in other words the aid kits distributed are equal to the amount required.

In relationship of the strategies used it can be concluded that when the amount of aid kits distributed are lower that the amount required, is because the initial donations of the actors involved are lower than the necessities which a circumstance that shouldn’t occur in the high-level disaster, therefore is necessary to create mechanism of cooperation between public and private sector or before a disaster be sure about possible suppliers and their capacities.

## 6 Conclusions

In this work we have analyzed the response capacity of humanitarian aid delivery in case of a disaster in the city of Bogotá-Colombia considering the local capacity (the same city), the neighboring cities of Bogotá which have a daily exchange of population and road network connections. In this sense a factor of analysis is the collaboration between stakeholders from neighboring cities and the national/international organizations. To evaluate the impact of collaboration in the response time, four strategies are proposed incorporating in the decision-making process conceptual elements found as relevant in the literature. Then, a simulation model based on system dynamics is proposed and parameterized into the Bogotá case study and its corresponding humanitarian policy, considering different levels of disasters.

Based on the results obtained by the four strategies proposed and taking into account the conceptual elements considered, we have defined a new strategy which contemplates the creation of join knowledge, by the use of a mathematical model to determine the optimal shipping policies of aid by each actor and transportation modes. The results show an improvement of the first 4 strategies proposed reducing the average response time even with lower values than those obtained for the first strategy (which have lower average response times).

With the proposed model it can be analyzed which type of donations are better to support the aid distribution in case of a disaster in Bogotá, physical or in cash. This can be analyzed from the results of the simulation given that the physical aids take time to be delivered affecting the response times of the subsystem. Also is important to consider that policy makers must develop agreements in different levels, local, regional, and national, to be able to have the capacity to deliver all the aid kits required to satisfy the affected population.

In future research, the proposed model can be extended including the evaluation of costs such as procurement cost, inventory holding cost, and operational costs with the aim to do a cost-effective analysis. Also, it can evaluate the impact of using framework agreements with private suppliers in the average response times.

Finally, a limitation of this work consists of the unavailability of a complete database regarding previous disasters in Bogotá. The last high magnitude earthquake in the capital city was presented on August 31, 1917. For this reason, it was not possible to compare the behavior with historical data and we parameterized the model with the current capacities.

This situation highlights the necessity for Bogotá of being prepared in case of a possible disaster and to identify its logistics capacities, possible strategies, collaborative agreements, among others with the aim to be able to meet the needs of the possible affected population.

Regarding the strategies, a limitation relies on the assumptions considered in the construction and modeling of the strategies, they can be validated with practitioners to check its applicability. Also, it can be considered additional elements of collaborative logistics concepts to design new strategies or to include new elements to the proposed ones.

## Declarations

### Author contribution statement

Diana C. Guzmán-Cortés: Conceived and designed the experiments; Performed the experiments; Analyzed and interpreted the data; Contributed reagents, materials, analysis tools or data; Wrote the paper.

Leonardo Gonzalez: Conceived and designed the experiments; Analyzed and interpreted the data; Contributed reagents, materials, analysis tools or data; Wrote the paper.

Carlos Franco, Ph. D; William Guerrero: Analyzed and interpreted the data; Contributed reagents, materials, analysis tools or data; Wrote the paper.

### Funding statement

This research did not receive any specific grant from funding agencies in the public, commercial, or not-for-profit sectors.

### Data availability statement

Data included in article/supp. material/referenced in article.

### Declaration of interest’s statement

The authors declare no conflict of interest.

### Additional information

No additional information is available for this paper.

## Appendix A Supplementary data

Supplementary Material_V2 Supplementary Material_V2
