# Supplementary material for: A simulation approach for collaborative humanitarian aid distribution management: the case of Bogotá city
Source: Heliyon. 2022 Nov 15;8(11):e11465. doi: 10.1016/j.heliyon.2022.e11465 (PMC9674513; doi:10.1016/j.heliyon.2022.e11465)
Supplement: Supplementary Material_V2 [file mmc1.docx]

**Supplementary Material**

**Appendix 1**

| Name | Type |
| --- | --- |
| ETS 1 | Aux/Const/Data |
| ETS 2 | Aux/Const/Data |
| ETS 3 | Aux/Const/Data |
| ETS 4 | Aux/Const/Data |
| ETS 5 | Aux/Const/Data |
| REQUIRED KITS | Aux/Const/Data |
| TIME STEP | Aux/Const/Data |
| TNA 5 | Aux/Const/Data |
| TSA 1 | Aux/Const/Data |
| TSA 2 | Aux/Const/Data |
| TSA 3 | Aux/Const/Data |
| TSA 4 | Aux/Const/Data |
| # Total Aircraft Cargo 1 ring | Aux/Const/Data |
| # Total Aircraft Cargo Local | Aux/Const/Data |
| # Total Aircraft Cargo Nat-Inter-NGO's | Aux/Const/Data |
| # Total Staff Aircraft 1 ring | Aux/Const/Data |
| # Total Staff Aircraft Local | Aux/Const/Data |
| # Total Staff Aircraft Nat-Inter-NGO's | Aux/Const/Data |
| # total trips per aircraft per hour 1 ring | Aux/Const/Data |
| # total trips per aircraft per hour Local | Aux/Const/Data |
| # total trips per aircraft per hour Nat-Inter-NGO's | Aux/Const/Data |
| # vehicles fleet type C2 (AP) | Aux/Const/Data |
| Airports Nat-Inter-NGO's | Aux/Const/Data |
| Average airport capacity (trips per hour) | Aux/Const/Data |
| Average heliport capacity (trips per hour) | Aux/Const/Data |
| Borrowed Airplane government-owned airlines | Aux/Const/Data |
| Cargo aircraft available Nat-Inter-NGO's | Aux/Const/Data |
| Heliports Nat-Inter-NGO's | Aux/Const/Data |
| Land vehicles available staff Nat- Inter- NGO's | Aux/Const/Data |
| Random VLC Nat-Inter-NGO's | Aux/Const/Data |
| Seed VLC Nat-Inter-NGO's | Aux/Const/Data |
| Staff Air Vehicles Available Nat-Inter-NGO's | Aux/Const/Data |
| Total airplane government-owned airlines | Aux/Const/Data |
| Trips available per cargo aircraft Nat-Inter-NGO's | Aux/Const/Data |
| Trips available per staff aircraft Nat-Inter-NGO's | Aux/Const/Data |
| Vehicles available LC Nat-Inter-NGO's | Aux/Const/Data |
| Air cargo vehicles available 1 ring | Aux/Const/Data |
| Air vehicles available staff 1 ring | Aux/Const/Data |
| Airplane Air Force | Aux/Const/Data |
| Airplane commercial airlines | Aux/Const/Data |
| Airport capacity | Aux/Const/Data |
| Airports 1° ring | Aux/Const/Data |
| borrowed air vehicle Air Force | Aux/Const/Data |
| Borrowed Airplane Air Force | Aux/Const/Data |
| Borrowed Airplane commercial airlines | Aux/Const/Data |
| Borrowed Cargo aircraft AF | Aux/Const/Data |
| Borrowed Cargo aircraft commercial airlines | Aux/Const/Data |
| Capacity available of Air Infrastructure | Aux/Const/Data |
| Cargo aircraft Air Force | Aux/Const/Data |
| Cargo aircraft commercial airlines | Aux/Const/Data |
| Heliports 1° ring | Aux/Const/Data |
| Heliports capacity | Aux/Const/Data |
| Land Cargo Vehicles available 1 ring | Aux/Const/Data |
| Land vehicles available staff 1 ring | Aux/Const/Data |
| Local Air cargo vehicles available | Aux/Const/Data |
| Local air vehicles available staff | Aux/Const/Data |
| Local Airports | Aux/Const/Data |
| Local Heliports | Aux/Const/Data |
| Local Land Cargo Vehicles Available | Aux/Const/Data |
| Local land vehicles available staff | Aux/Const/Data |
| Random borrowed air vehicle Air Force | Aux/Const/Data |
| Random borrowed air vehicle commercial airlines | Aux/Const/Data |
| Random Borrowed Cargo aircraft AF | Aux/Const/Data |
| Random borrowed Cargo aircraft commercial airlines | Aux/Const/Data |
| Random borrowed staff air vehicle | Aux/Const/Data |
| Seed borrowed air vehicle commercial airlines | Aux/Const/Data |
| Seed Borrowed Cargo aircraft AF | Aux/Const/Data |
| Seed Borrowed Cargo aircraft commercial airlines | Aux/Const/Data |
| Seed borrowed staff air vehicle | Aux/Const/Data |
| Switch disaster level | Aux/Const/Data |
| Total trips available per cargo aircraft | Aux/Const/Data |
| Total trips available per staff aircraft | Aux/Const/Data |
| Trips available per cargo aircraft 1 ring | Aux/Const/Data |
| Trips available per cargo aircraft Local | Aux/Const/Data |
| Trips available per staff aircraft 1 ring | Aux/Const/Data |
| Trips available per staff aircraft Local | Aux/Const/Data |
| # trips per hour land cargo vehicle 1 ring | Aux/Const/Data |
| # trips per hour land cargo vehicle Local | Aux/Const/Data |
| # trips per hour land cargo vehicle Nat-Inter-NGO's | Aux/Const/Data |
| % Air Infraestructure 1 ring | Aux/Const/Data |
| % Air Infraestructure Nat-Inter-NGO's | Aux/Const/Data |
| % Local Air Infrastructure | Aux/Const/Data |
| Air Infraestructure Capacity Available Nat-Inter-NGO's | Aux/Const/Data |
| Capacity land cargo Nat-Inter-NGO's | Aux/Const/Data |
| Capacity staff Nat-Inter-NGO's Land | Aux/Const/Data |
| Conditional Capacity Air Cargo Nat-Inter-NGO's | Aux/Const/Data |
| Conditional Capacity Staff Nat-Inter-NGO's Air | Aux/Const/Data |
| Delay Air Cargo Nat-Inter-NGO's | Aux/Const/Data |
| Delay Land Cargo Nat-Inter-NGO's | Aux/Const/Data |
| Delay staff Nat-Inter-NGO's Air | Aux/Const/Data |
| Delay staff Nat-Inter-NGO's Land | Aux/Const/Data |
| Desaly staff call Nat-Inter-NGO's | Aux/Const/Data |
| Initial Kits Nat-Inter-NGO's | Aux/Const/Data |
| Initial Quantity Kits Nat-Inter-NGO's | Aux/Const/Data |
| Random Initial Staff Nat-Inter-NGO's | Aux/Const/Data |
| Random Nat-Inter-NGO's | Aux/Const/Data |
| Random seed delay call Nat-Inter-NGO's | Aux/Const/Data |
| Seed delay aid call Nat-Inter-NGO's | Aux/Const/Data |
| Seed donation Nat-Inter-NGO's | Aux/Const/Data |
| Seed Initial Staff Nat-Inter-NGO's | Aux/Const/Data |
| Seed staff call Nat-Inter-NGO's | Aux/Const/Data |
| Table % infraestructure Nat-Inter-NGO's X | Aux/Const/Data |
| Table % infraestructure Nat-Inter-NGO's Y | Aux/Const/Data |
| Table % Local Air Infrastructure X | Aux/Const/Data |
| Table % Local Air Infrastructure Y | Aux/Const/Data |
| Table Initial Kits Nat-Inter-NGO's | Aux/Const/Data |
| Air Infraestructure Capacity Available 1 ring | Aux/Const/Data |
| Air Infraestructure Disruption | Aux/Const/Data |
| Average capacity Air Vehichle Staff | Aux/Const/Data |
| Average capacity Air vehicle Cargo | Aux/Const/Data |
| Average capacity Land Vehichle Staff | Aux/Const/Data |
| Average capacity Land vehicle Cargo | Aux/Const/Data |
| Capacity Land Cargo 1 ring | Aux/Const/Data |
| Capacity Land Cargo Local | Aux/Const/Data |
| Capacity Local Staff Land | Aux/Const/Data |
| Capacity Staff 1 ring Land | Aux/Const/Data |
| Conditional Capacity Air Cargo 1 ring | Aux/Const/Data |
| Conditional Capacity Air Cargo Local | Aux/Const/Data |
| Conditional capacity local staff Air | Aux/Const/Data |
| Conditional capacity staff 1 ring Air | Aux/Const/Data |
| Delay Air Cargo 1 ring | Aux/Const/Data |
| Delay Air Cargo Local | Aux/Const/Data |
| Delay Land Cargo 1 ring | Aux/Const/Data |
| Delay Land Cargo Local | Aux/Const/Data |
| Delay Local Staff Air | Aux/Const/Data |
| Delay Local Staff Land | Aux/Const/Data |
| Delay staff 1 ring Air | Aux/Const/Data |
| Delay staff 1 ring Land | Aux/Const/Data |
| Delivery Efficiency Staff | Aux/Const/Data |
| Initial Kits 1 ring | Aux/Const/Data |
| Initial Kits population 1 ring | Aux/Const/Data |
| Initial Local Kits | Aux/Const/Data |
| Initial Local Staff | Aux/Const/Data |
| Initial Quantity Kits 1 ring | Aux/Const/Data |
| Initial Quantity Local Kits | Aux/Const/Data |
| Initial Staff 1 ring | Aux/Const/Data |
| Percentage Satisfied Demand | Aux/Const/Data |
| Random delay aid call 1 ring | Aux/Const/Data |
| Random delay aid call Local | Aux/Const/Data |
| Random delay local staff call | Aux/Const/Data |
| Random delay staff call 1 ring | Aux/Const/Data |
| Random donation 1 ring | Aux/Const/Data |
| Random Local Donations | Aux/Const/Data |
| Roadways Disruption | Aux/Const/Data |
| Seed delay aid call 1 ring | Aux/Const/Data |
| Seed delay humanitarian aid call Local | Aux/Const/Data |
| Seed delay local staff call | Aux/Const/Data |
| Seed delay staff call 1 ring | Aux/Const/Data |
| Seed donation 1 ring | Aux/Const/Data |
| Seed Local Donations | Aux/Const/Data |
| Table Air Infraestructure Disruption X | Aux/Const/Data |
| Table Air Infraestructure Disruption Y | Aux/Const/Data |
| Table initial kits Local population X | Aux/Const/Data |
| Table initial kits Local population Y | Aux/Const/Data |
| Table Roadways Disruption X | Aux/Const/Data |
| Table Roadways Disruption Y | Aux/Const/Data |
| Units Adjustment Factor | Aux/Const/Data |
| % affected population | Aux/Const/Data |
| Affected population | Aux/Const/Data |
| AVERAGE RESPONSE TIME | Aux/Const/Data |
| Factor population per KIT | Aux/Const/Data |
| N1 | Aux/Const/Data |
| N2 | Aux/Const/Data |
| N3 | Aux/Const/Data |
| N4 | Aux/Const/Data |
| N5 | Aux/Const/Data |
| Random | Aux/Const/Data |
| Seed afected population | Aux/Const/Data |
| Table N1 X | Aux/Const/Data |
| Table N1 Y | Aux/Const/Data |
| Table N2 X | Aux/Const/Data |
| Table N2 Y | Aux/Const/Data |
| Table N3 X | Aux/Const/Data |
| Table N3 Y | Aux/Const/Data |
| Table N4 X | Aux/Const/Data |
| Table N4 Y | Aux/Const/Data |
| Table N5 X | Aux/Const/Data |
| Table N5 Y | Aux/Const/Data |
| Time | Aux/Const/Data |
| ELA 1 | Level |
| ELA 2 | Level |
| ELA 3 | Level |
| ELA 4 | Level |
| ELA 5 | Level |
| Air cargo vehicles Nat-Inter-NGO's assigned 5 | Level |
| Staff air vehicles assigned Nat-Inter-NGO's 5 | Level |
| Staff land vehicles assigned Nat- Inter- NGO's 5 | Level |
| Vehicles land cargo assigned Nat-Inter-NGO's 5 | Level |
| Air Cargo vehicles 1 ring assigned 5 | Level |
| Airport assigned 5 | Level |
| Heliports assigned 5 | Level |
| Local Air cargo vehicles assigned 5 | Level |
| Local land cargo vehicles assigned 5 | Level |
| Local staff air vehicles assigned 5 | Level |
| Local staff land vehicles assigned 5 | Level |
| Staff air vehicles assigned 1 ring 5 | Level |
| Staff land vehicles assigned 1 ring 5 | Level |
| Vehicles land cargo assigned 1 ring 5 | Level |
| Inventory Level kits Nat-Inter-NGO's | Level |
| Level Kits Nat-Inter-NGO's | Level |
| Level random initial staff Nat-Inter-NGO's | Level |
| Level random Nat-Inter-NGO's | Level |
| Level Staff Nat-Inter-NGO's with delay | Level |
| Level Staff Nat-Inter-NGO's | Level |
| Delivered kits level | Level |
| Inventory Level Kits 1 ring | Level |
| Inventory Level Local Kits | Level |
| Level Kits 1 ring | Level |
| Level Local Kits | Level |
| Level Local Staff | Level |
| Level Local Staff with delay | Level |
| Level random donation | Level |
| Level random donation 1 ring | Level |
| Level Satff 1 ring | Level |
| Level staff 1 ring with delay | Level |
| Level total staff sent | Level |
| Total Level food kit to affected population | Level |
| Affected level N1 | Level |
| Affected level N2 | Level |
| Affected level N3 | Level |
| Affected level N4 | Level |
| Affected level N5 | Level |
| AFFECTED POPULATION LEVEL | Level |
| Response time level | Level |
| SERVED POPULATION LEVEL | Level |
| ERA 1 | Rates |
| ERA 2 | Rates |
| ERA 3 | Rates |
| ERA 4 | Rates |
| ERA 5 | Rates |
| Air cargo vehicles rate Nat-Inter-NGO's assigned 5 | Rates |
| Air cargo vehicles rate Nat-Inter-NGO's returned 5 | Rates |
| Land cargo vehicles rate Nat- Inter- NGO's ONGs Assinged 5 | Rates |
| staff air vehicles rate assigned Nat-Inter-NGO's 5 | Rates |
| Staff land vehicles rate assigned Nat- Inter- NGO's 5 | Rates |
| Staff land vehicles rate returned Nat- Inter- NGO's 5 | Rates |
| Tstaff air vehicles rate returned Nat-Inter-NGO's 5 | Rates |
| Air Cargo vehicles rate 1 ring assigned 5 | Rates |
| Air cargo vehicles rate 1 ring returned 5 | Rates |
| Land cargo vehicles rate 1 ring assigned 5 | Rates |
| Local air cargo vehicles rate assigned 5 | Rates |
| Local air cargo vehicles rate returned 5 | Rates |
| Local land cargo vehicles rate assigned 5 | Rates |
| Local land cargo vehicles rate returned 5 | Rates |
| local staff air vehicles rate assigned 5 | Rates |
| local staff air vehicles rate returned 5 | Rates |
| local staff land vehicles rate assigned 5 | Rates |
| local staff land vehicles rate returned 5 | Rates |
| Rate of airport assigned 5 | Rates |
| Rate of airport returned 5 | Rates |
| Rate of Heliports assigned 5 | Rates |
| Rate of Heliports returned 5 | Rates |
| staff air vehicles rate assigned 1 ring 5 | Rates |
| staff air vehicles rate returned 1 ring 5 | Rates |
| staff land vehicles rate assigned 1 ring 5 | Rates |
| staff land vehicles rate returned 1 ring 5 | Rates |
| Vehicles land cargo rate returned 1 ring 5 | Rates |
| Vehicles land cargo rate returned 5 | Rates |
| Arrival rate kits air cargo Nat-Inter-NGO's | Rates |
| Arrival rate kits land cargo Nat-Inter-NGO's | Rates |
| Arrival rate staff Nat-Inter-NGO's Air | Rates |
| Arrival rate staff Nat-Inter-NGO's Land | Rates |
| Dispatch rate staff Nat-Inter-NGO's Air | Rates |
| Dispatch rate staff Nat-Inter-NGO's Land | Rates |
| Distribution rate kits Nat-Inter-NGO's Air Cargo | Rates |
| Distribution rate kits Nat-Inter-NGO's Land Cargo | Rates |
| Output rate kits Nat-Inter-NGO's | Rates |
| Output rate staff Nat-Inter-NGO's | Rates |
| Procurement Rate Nat-Inter-NGO's | Rates |
| Procurement rate staff Nat-Inter-NGO's | Rates |
| Rate random initial staff Nat-Inter-NGO's | Rates |
| Rate random Nat-Inter-NGO's | Rates |
| Arrival rate kits air cargo 1 ring | Rates |
| Arrival rate kits land cargo 1 ring | Rates |
| Arrival rate Local Kits Air cargo | Rates |
| Arrival rate Local Kits Land cargo | Rates |
| Arrival rate Local Staff Air | Rates |
| Arrival rate Local Staff Land | Rates |
| Arrival rate staff 1 ring Air | Rates |
| Arrival rate staff 1 ring Land | Rates |
| Delivery rate food kits | Rates |
| Dispatch rate local staff air | Rates |
| Dispatch rate local staff land | Rates |
| Dispatch rate staff 1 ring Air | Rates |
| Dispatch rate staff 1 ring Land | Rates |
| Distribution rate kits 1 ring air cargo | Rates |
| Distribution rate kits 1 ring land cargo | Rates |
| Distribution Rate Local Kits Air cargo | Rates |
| Distribution Rate Local Kits Land Cargo | Rates |
| Output rate kits 1 ring | Rates |
| Output rate local kits | Rates |
| Output rate local staff | Rates |
| Output rate staff 1 ring | Rates |
| Procurement rate kits 1 ring | Rates |
| Procurement rate local kits local | Rates |
| Procurement rate local staff | Rates |
| Procurement rate staff 1 ring | Rates |
| Rate random 1 ring | Rates |
| Rate random donation | Rates |
| Affected population rate | Rates |
| Affected population rate N1 | Rates |
| Affected population rate N2 | Rates |
| Affected population rate N3 | Rates |
| Affected population rate N4 | Rates |
| Affected population rate N5 | Rates |
| Attention rate to population | Rates |
| Response time rate | Rates |
